# Transcriptional Host Responses to Infection with *Streptococcus suis* in a Porcine Precision-Cut Lung Slice Model: Between-Strain Differences Suggest Association with Virulence Potential

**DOI:** 10.3390/pathogens13010004

**Published:** 2023-12-19

**Authors:** Yenehiwot Berhanu Weldearegay, Louise Brogaard, Andreas Nerlich, Désirée Schaaf, Peter M. H. Heegaard, Peter Valentin-Weigand

**Affiliations:** 1 Institute for Microbiology, Department of Infectious Diseases, University of Veterinary Medicine Hannover, 30173 Hannover, Germany; yeneb.welde@gmx.de (Y.B.W.); andreas.nerlich@fu-berlin.de (A.N.); desiree.schaaf@tiho-hannover.de (D.S.); 2 Department of Biotechnology and Biomedicine, Section for Protein Science and Biotherapeutics, Technical University of Denmark, 2800 Kongens Lyngby, Denmark; loun@dtu.dk (L.B.); pmhh@dtu.dk (P.M.H.H.); 3 Department of Veterinary Medicine, Veterinary Centre for Resistance Research (TZR), Freie Universität Berlin, 14163 Berlin, Germany; 4 Department of Health Technology, Experimental & Translational Immunology, Technical University of Denmark, 2800 Kongens Lyngby, Denmark

**Keywords:** *Streptococcus suis*, gene expression, cytokines, PCLS, virulence

## Abstract

*Streptococcus suis* is a porcine and zoonotic pathogen in the upper respiratory tract, expressing different capsular serotypes and virulence-associated factors. Given its genomic and phenotypic diversity, the virulence potential of *S. suis* cannot be attributed to a single factor. Since strong inflammatory response is a hallmark of *S. suis* infection, the objective of this study was to investigate the differences in transcriptional host responses to two serotype 2 and one serotype 9 strains. Both serotypes are frequently found in clinical isolates. We infected porcine precision-cut lung slices (PCLSs) with two serotype 2 strains of high (strain S10) and low (strain T15) virulence, and a serotype 9 strain 8067 of moderate virulence. We observed higher expression of inflammation-related genes during early infection with strains T15 and 8067, in contrast to infection with strain 10, whose expression peaked late. In addition, bacterial gene expression from infected PCLSs revealed differences, mainly of metabolism-related and certain virulence-associated bacterial genes amongst these strains. We conclude that the strain- and time-dependent induction of genes involved in innate immune response might reflect clinical outcomes of infection in vivo, implying rapid control of infection with less virulent strains compared to the highly virulent strain S10.

## 1. Introduction

*Streptococcus suis* is a pathobiont of the upper respiratory tract of pigs causing septicemia, meningitis, arthritis, and pneumonia, mainly in pigs [1]. *S. suis* is responsible for a substantial economic burden and impaired animal welfare in pig production worldwide. It also poses a zoonotic threat, although except for two larger outbreaks of *S. suis* infection in humans in China in 1998 and 2005, human cases are sporadic and require close contact between humans and pigs or pig products to occur [2,3]. *S. suis* is a highly diverse bacterial species, comprising over 29 serotypes and several hundred sequence types [1,4]. A multitude of different factors have been identified or suggested to be important for the virulence of *S. suis* [5,6,7]. The most frequently addressed virulence-associated factors in the literature are the capsular polysaccharide (CPS), pore-forming cytolysin suilysin (*sly*), extracellular factor (*epf*), and muramidase-released protein (*mrp*) [8,9,10,11,12,13,14,15,16]. However, considering the large diversity of the species, the virulence of a given strain cannot be attributed solely to one single factor. In the effort to describe the virulence potential of *S. suis* strains, an alternative could be to focus on the host response they induce during infection, thus identifying host responses associated with different virulence levels, which may explain the differences in virulence observed in vivo.

The innate immune response against *S. suis* has been investigated in various in vivo and in vitro models [17,18,19,20,21,22,23], demonstrating the induction of pro- and anti-inflammatory cytokine production as well as chemokine production on *S. suis* infection. This includes the upregulation of *IL1A*, *IL1B*, and *IL1RN* transcription in nasal mucosal swabs after *S. suis* strain 10 challenge [17] and upregulation of *IL1B*, *IL6*, *TNF*, and *CXCL8* transcription in the lung after *S. suis* strain SC19 challenge [23], both of which are serotype 2 strains. However, there is a general lack of insight into how *S. suis* strains of varying virulence shape the host response, and to which degree and in what way this host response might correlate with *S. suis* virulence potential. The innate immune system constitutes the first line of defense during infection, and differential induction of innate immune mechanisms by *S. suis* strains with varying virulence potential might therefore play a role in shaping the pathogenesis of *S. suis* infection. Thus, we hypothesized that investigating the early innate immune response induced by *S. suis* strains of varying virulence may reveal the mechanisms driving the severe clinical outcomes of infection with highly virulent strains and how these mechanisms differ from the ones induced by low or non-virulent strains. This could ultimately aid the identification of bacterial functions and factors that are defining the virulence potential of a given *S. suis* strain. 

In compliance with the 3R guidelines (replacement, reduction, and refinement) [24], the ex vivo precision-cut lung slice (PCLS) model has previously been applied to study host–pathogen interactions in swine [25], goats, and cattle [26]. Moreover, induction of the innate immune response at the transcriptional level has also previously been demonstrated in a mouse PCLS model after infection with rhinovirus [27]. It is a three-dimensional organ model, which reflects the in vivo cellular architecture of the lung, thus representing a valid in vitro mimic of the infected lung, allowing the study of the early innate immune response induced by tissue-resident immune cells of the lung during *S. suis* infection. The cellular makeup of the lung, and thus also the PCLS model, comprises numerous cell types that are important contributors in the host response to bacterial infection, including alveolar macrophages, dendritic cells, neutrophils, T-cells, B-cells, epithelial cells, fibroblasts, endothelial cells, and lymphatic cells [28,29,30,31]. 

In the present study, the porcine PCLS model is used for simultaneous investigation of host and pathogen transcriptional dynamics using high-throughput RT-qPCR, facilitating parallel quantification of porcine and bacterial gene expression in the same sample, as described previously [32]. The moderately virulent *S. suis* serotype 9 strain 8067, non-virulent serotype 2 strain T15, and the highly virulent serotype 2 strain 10 (S10) [17,33] were investigated for their ability to induce an innate immune response in the PCLS model. Concurrently, the bacterial expression of virulence and metabolism-associated genes during infection was assessed. Our results demonstrated a distinct pro-inflammatory cytokine as well as chemokine response specific to infection with the highly virulent S10 strain and different from the response induced by the low and non-virulent strains. Regarding bacterial gene expression, genes involved in carbohydrate transport and metabolism were generally most highly expressed in the moderately virulent strain 8067, whereas virulence-associated genes were highly expressed at early time points during S10 infection.

## 2. Materials and Methods

### 2.1. Bacterial Strains and Culture Conditions

*S. suis* serotype 2 (strains S10 and T15) and serotype 9 (strain 8067) were used. Bacteria were grown in Todd Hewitt broth (THB) medium until the late exponential phase in a total volume of 20 mL until optical density at 600 nm (OD_600_) reached 1.0–1.2. Then, 10 mL of culture was centrifuged at 4000× *g* for 10 min at 4 °C, the supernatant was discarded, and the pellet was resuspended in 1 mL of THB medium with 15% glycerol. The suspension was aliquoted in 50 µL, snap frozen in liquid nitrogen, and stored at −80 °C. The bacterial titer of the infection cryostocks was determined after storage at −80 °C.

### 2.2. Preparation of PCLS

For each batch of PCLS preparation, a minimum of three lungs from healthy slaughtered pigs from a local abattoir in Laatzen, Germany, were brought on ice to the laboratory. On average, from a single batch of PCLS preparation, approximately 150 good quality PCLS could be obtained. The results included in this study represent a minimum of three independent experiments. 

PCLS were prepared according to the method described previously [26] with minor modifications. Cranial, cardiac, and accessory lobes were separated and filled with 1.5% low-melting agarose (GERBU, Heidelberg, Germany) in Roswell Park Memorial Institute (RPMI) medium (Sigma-Aldrich, Taufkirchen, Germany), which had been brought to boiling point to dissolve the agarose completely and cooled to 37 °C. The agarose-filled lobes were kept on ice until the agarose solidified, making sectioning easier. Cylindrical sections with bronchi/bronchioles in the center were punched out with a tissue coring tool and sliced with a Krumdieck tissue slicer (model MD 4000-01; TSE Systems, Chesterfield, MO, USA) filled with cold RPMI medium of 300–400 µm thickness. The slices were placed in 80 mL of RPMI 1640 medium supplemented with antibiotics and antimycotics (with final concentration of 10 mL/L of penicillin/streptomycin stock solution containing 5000 U/mL penicillin and 5000 U/mL streptomycin, 50 mg/l kanamycin, 2.5 mg/L amphotericin B, and 1 mg/L clotrimazole). The medium with the above supplements was designated RPMI-1. Slices were then bubbled with a normoxic gas mixture at 37 °C for two hours to remove the agarose, as per the method described by Paddenberg et al. [34]. At the end of the bubbling, individual slices were transferred to 24-well plates with 1 mL of RPMI-1. This process removed ~95% of the agarose. Slices were further incubated at 37 °C with 5% CO_2_ overnight in RPMI-1. On the following day (one day before infection), ciliary activity as a parameter of PCLS viability was monitored using a Leica DMi1 light microscope (Leica, Wetzlar, Germany), according to the method described by Punyadarsaniya et al. [35]. Slices with a ciliary activity of more than 95% were washed twice with phosphate-buffered saline (PBS) (Sigma-Aldrich) and 1 mL of RPMI 1640 medium without any supplement, designated RPMI-2, was added to each slice. RPMI-2 was used for the remainder of the experiment.

### 2.3. Infection of PCLS

PCLS were washed twice with PBS and infected with 10^7^ CFU in 1 mL of RPMI-2, followed by incubation in a humidified incubator at 37 °C with 5% CO_2_. Uninfected controls were incubated with RPMI-2 medium only. Slices were collected for RNA extraction and immunofluorescence analysis at 0 (immediately after infection), 4, 8, and 24 h post-infection (hpi). For RNA extraction, two slices were kept in 500 µL of RNAlater RNA Stabilization Reagent (Qiagen, Hilden, Germany), incubated overnight at 4 °C, and then transferred to −20 °C until extraction. Medium from the infected and uninfected PCLS at all time points were individually collected for enzyme-linked immunosorbent assay (ELISA) and centrifuged at 4000× *g* at 4 °C for 10 min. The supernatants were then stored at −80 °C until analysis. For immunofluorescence analysis, samples were washed three times with PBS and fixed with 4% paraformaldehyde in PBS for 24–48 h at room temperature, washed three times with PBS, and transferred to 70% ethanol until paraffin embedding.

### 2.4. Isolation of Porcine and Bacterial RNA

RNA was extracted according to the method described by Niehof et al. [36]. Two slices were used for each extraction. Briefly, slices were removed from the RNAlater, and excess RNAlater was removed using tissue paper. The slices were transferred to 2 mL screw-capped tubes with Lysing Matrix Z (MP Biomedicals, Irvine, CA, USA) and 0.1 mm zirconium beads (Carl Roth, Karlsruhe, Germany). RLT buffer (400 µL) from Qiagen RNeasy Mini kit was added, and slices were homogenized using FastPrep-24™ 5G (MP Biomedicals) twice at level 5 for 45 s with 2 min cooling on ice during intervals. Chloroform and phenol, each 200 µL, were added and mixed for 30 s by inverting. Samples were centrifuged at 12,000× *g* at 4 °C for 5 min. The aqueous phase was transferred into a new 1.5 mL tube, where 400 µL of chloroform/isoamyl alcohol (49:1) was added and mixed for 30 s, followed by 5 min centrifugation as above. The aqueous phase was transferred to a new 1.5 mL tube and half a volume of isopropanol was added to precipitate the RNA. From this point, the MagMax kit (Thermo Fisher Scientific, Waltham, MA, USA) protocol was followed according to the manufacturer’s instructions. RNA yield and purity were assessed using a NanoDrop ND-1000 UV spectrophotometer (Thermo Fisher Scientific). The mean A260/280 ratio for all RNA samples was 2.0 and mean A260/230 ratio was 1.5; RNA yield ranged from 54 ng/µL to 593 ng/µL with a mean yield of 213 ng/µL. Extracted RNA was stored at −80 °C until further analysis. Residual beads left over from the RNA isolation procedure interfered with the measurement of RNA integrity numbers (RIN) by means of on-chip gel electrophoresis (Figures S1–S7). A comprehensive pilot study in which a clean-up procedure was applied to remove residual beads confirmed that RNA from PCLS obtained by the method described here was of excellent quality; however, a large amount of the RNA was lost during the clean-up procedure and the qPCR results obtained from cDNA made from cleaned-up and non-cleaned-up RNA were comparable. Clean-up was thus not performed on the RNA included in the present study to avoid unnecessary loss of material.

### 2.5. Reverse Transcription and Pre-Amplification of cDNA

cDNA was prepared using the QuantiTect Reverse Transcription kit (Qiagen) according to the manufacturer’s instructions, including initial DNase treatment with gDNA Wipeout Buffer (Qiagen). A total of 450 ng RNA was used in each synthesis. No-RT controls were included where the reverse transcriptase had been replaced with RNase-free water. Two cDNA preparations were synthesized from each individual RNA sample (technical replicates). cDNA samples were pre-amplified using TaqMan PreAmp Master Mix (Applied Biosystems, Waltham, MA, USA) and a combined pool of all porcine and bacterial primer pairs (each primer pair at 200 nM) were included in the following qPCR. Pre-amplification was carried out with 20 amplification cycles. Residual primers were subsequently digested by Exonuclease I treatment (New England Biolabs, Ipswitch, MA, USA).

### 2.6. High-Throughput qPCR

Porcine and bacterial gene expression were analyzed simultaneously in each PCLS sample using the Biomark HD real-time instrument (Fluidigm, South San Francisco, CA, USA) and 2X TaqMan Gene Expression Master Mix (Applied Biosystems), EvaGreen Dye 20X (Biotium, Fremont, CA, USA), 20X DNA Binding Dye (Fluidigm), 2X Assay Loading Reagent (Fluidigm), and in-house designed qPCR primer pairs (Figures S1–S7). A panel of 49 porcine and 47 bacterial assays were combined to fill the 96.96 Dynamic Array IFC chip (Fluidigm), accommodating 96 assays and 96 samples. A total of four 96.96 Dynamic Array IFC chips were required to accommodate the total number of samples to be analyzed, which included the individual pre-amplified cDNA samples, no-RT controls, no-template controls (NTCs), inter-plate calibrators, and the individual dilution series for primer efficiency determination in the mock and in strains 8067-, T15-, and S10-infected PCLS, respectively. Porcine genes representing a broad spectrum of innate immune functions potentially affected by *S. suis* infection in the PCLS model were chosen for analysis, including genes related to Toll-like receptor signaling, transcription factors, pro- and anti-inflammatory cytokines and other inflammation markers, chemokines, tight junction integrity, and airway surface liquid homeostasis. With regard to bacterial gene expression, focus was on gene coding for previously reported known or putative virulence-associated factors as well as a selection of metabolism-related genes. Porcine primers were (whenever possible) designed to span intron/exon borders to ensure the amplification of cDNA alone and not gDNA. Bacterial primers were designed based on the *S. suis* BM407 genome (serotype 2) followed by BLAST analysis to ensure that (1) the primer sequences were unique in the *S. suis* genome, and (2) the primer sequences matched *S. suis* serotype 9 genomes, so that expression of the genes of interest could be quantified using the same primer pair in both the serotype 2 and 9 strains used in the present study. Primer pairs for porcine and bacterial targets were defined using the online Primer3 software (v. 0.4.0, http://bioinfo.ut.ee/primer3-0.4.0/, accessed on 8 and 23 March 2018). Thirty-five cycles of qPCR amplification were carried out followed by melting curve analysis.

### 2.7. qPCR Data Analysis

All amplification and melting curves were inspected visually using the Fluidigm Real-Time PCR Analysis software (v. 4.1.3). This software was likewise used to evaluate dilution series in order to determine qPCR primer efficiencies. The GenEx Enterprise software (v. 7) was used for inter-plate calibration, qPCR efficiency correction, evaluation of porcine and bacterial reference genes using the geNorm [37] and NormFinder [38] algorithms, and subsequent normalization with suitable reference genes (*B2M*, *HPRT1*, *PPIA*, *YWHAZ*, *RPL13A* for normalization of porcine gene expression; *rpoB*, *topA*, *gyrB*, *dnaK*, *adk*, *gmk* for normalization of bacterial gene expression). Cq (quantification cycle) values were converted to a linear scale by computing relative quantities. Briefly, for each assay, the sample with the lowest expression of that particular gene, i.e., highest Cq, was scaled to 1 and all other samples, i.e., across all three strains within the assay, were scaled relative to this, giving them relative expression values higher than 1. Data analysis was performed only for 4, 8, and 24 hpi groups for bacterial gene expression (low amounts of bacterial mRNA in 0 hpi samples made quantification unreliable), whereas porcine gene expression was evaluated at all time points.

### 2.8. Immunofluorescence Staining

Individual slices, at least two per time point and per experiment, were fixed with 4% paraformaldehyde (PFA) in PBS for 24–48 h in a 24-well plate. Samples were washed with PBS, transferred to 70% ethanol, and embedded in paraffin blocks. A rotary microtome (Leitz 1512; Leitz, Oberkochen, Germany) was used to section the paraffin blocks into slices of 3–4 µm, which were mounted on Histobond microscope slides. Quality control was performed using hematoxyline and eosin (H&E) staining. Immunofluorescence (IF) staining was performed according to the methods described in our previous studies [26,39,40] with some modifications. Paraffin-embedded slices were first deparaffinized using Roti Histol and rehydrated with a series of ethanol concentrations (100%, 95%, 70% EtOH) and finally with PBS. For antigen retrieval, samples were heated in 10 mM sodium citrate (pH 6.0) in a pressure cooker for 10 min. After washing samples once with PBS, non-specific sites were blocked with 1% bovine serum albumin (BSA) (Carl Roth) in PBS. All antibodies were diluted in PBS with 1% BSA. The primary antibody against *S. suis* (in-house made polyclonal anti-*S. suis* antiserum raised in rabbits by immunization with a whole-cell preparation of strain S10, diluted 1:500) was used in combination with a goat anti-rabbit IgG (H+L)-Alexa Fluor 488 secondary antibody (1:1000, ThermoFischer Scientific). The β-tubulin of the cilia was stained using mouse monoclonal anti-β-tubulin−Cy3 antibody (1:500, Sigma-Aldrich). The nuclei were stained using 4’,6-diamidino-2-phenylindole (DAPI) (0.5 µg/mL in PBS, Cell Signaling Technology, Beverly, MA, USA). Samples were finally embedded in ProLong Gold Antifade Reagent (Cell Signaling Technology). Stained samples were visualized using a Nikon Eclipse Ti-S inverted fluorescence microscope equipped with Plan Fluor 10x/0.30 DIC and Plan Fluor 60x/0.50–1.25 Oil Iris objectives and a Nikon Digital Sight DS-Qi1Mc camera (Nikon, Tokyo, Japan). Images were captured with NIS-Elements software BR version 4.51.01. Image contrast and brightness were adjusted using ImageJ Version 1.51q software (National Institute of Health, Bethesda, MD, USA).

### 2.9. ELISA

IL-1β and IL-6 were quantified in supernatants (undiluted) collected from PCLS cultivation with strains 8067, T15, S10, as well as mock-infected PCLS. Analysis was performed using the sandwich ELISA kits DY681 (IL-1β) and DY686 (IL-6) (R&D Systems, Abingdon, UK) following the manufacturer’s instructions. Briefly, goat anti-pig IL-1β/IL-6 antibody was used for capture and biotinylated goat anti-pig IL-1β/IL-6 for detection. A porcine IL-1β/IL-6 standard was included for quantification. The development of plates was carried out according to the manufacturer’s instructions using tetramethylbenzidine (TMB) peroxide color substrate (Kementec, Taastrup, Denmark). The detection limit for both ELISAs was 31.25 pg/mL; for all samples below the detection limit, the detection limit value was applied for statistical analysis.

### 2.10. Statistical Analysis and Data Visualization

Gene expression data were log_2_ transformed prior to statistical analyses and principal component analysis (PCA); the normal distribution of log_2_ transformed data was confirmed using the Shapiro–Wilk test. The statistical significance of differential porcine gene expression in post-infection groups relative to the appertaining 0 hpi group was determined using Student’s *t*-test (*p* < 0.05), applying the Benjamini–Hochberg procedure to control the false discovery rate (FDR) (Microsoft Excel, version 2016). The statistical significance of differential bacterial gene expression at 4, 8, and 24 hpi, respectively, was determined using one-way ANOVA (*p* < 0.05, applying the Benjamini–Hochberg procedure to control the FDR) (Microsoft Excel). The statistical significance of difference in protein concentrations for IL-1β and IL-6 in PCLS supernatants was calculated for all post-infection groups relative to the appertaining 0 hpi group using the Mann–Whitney U test (GraphPad Prism v. 8.4.3). All graphical representations of transcriptional and ELISA results were made using GraphPad Prism and depict group means and 95% confidence intervals (CI). Principal component analysis (PCA) of gene expression data was performed using the GenEx Enterprise software (v. 7) for selected groups of porcine genes, i.e., genes related to pattern recognition receptors (PRRs) and transcription factors (*TLR2*, *TLR4*, *TLR6*, *MYD88*, *NFKB1*, *NFKBIA*, *IRF1*, *JUN*) and genes related to the inflammatory response (*IL1A*, *IL1B*, *IL6*, *IL18*, *TNF*, *CCL2*, *CCL3*, *CXCL8*, *CXCL10*, *CSF2*, *PTGS2*, *IL10*, *IL1RN*, *SAA*, *PTX3*). PCA was performed including all samples from all three post-infection time points. 

## 3. Results

### 3.1. Growth, Adherence, and Colonization of S. suis in Porcine PCLS

The PCLS were infected with 10^7^ CFU of *S. suis* of each strain. The bacterial number was determined at 0, 4, 8, and 24 hpi from the supernatants, demonstrating a comparable growth rate of the three strains in the presence of PCLS (Figure S1). 

**Figure 1:**
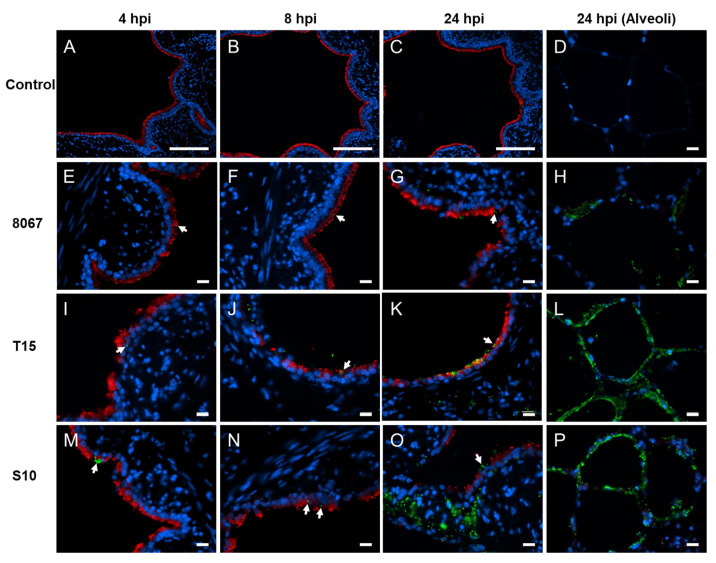
Colonization of porcine PCLS with *S. suis* as visualized by immunofluorescence staining. Uninfected controls (**A**–**D**), infected with strains 8067 (**E**–**H**), T15 (**I**–**L**), and S10 (**M**–**P**). Adhesion of the bacteria at 4 (**E**,**I**,**M**), 8 (**F**,**J**,**N**), and 24 (**G**,**K**,**O**) hpi to the ciliated cells followed by colonization of the alveolar epithelium by all strains at 24 hpi (**H**,**L**,**P**). Red: β-tubulin; green: *S. suis*; white arrows: *S. suis* attached to the cilia; blue: nuclei. Scale bars: for (**A**–**C**), 100 µm, and for the rest, 10 µm.

Immunofluorescence staining of the infected tissue slices revealed the adherence and/or colonization of the bacteria mainly on the ciliated cells (Figure 1) during the early stages of infection (Figure 1E,F,I,J,M,N). As the infection proceeded, bacteria were still observed on the ciliated cells (Figure 1G,K,O) but the majority were associated with the alveolar epithelium (Figure 1H,L,P) and the peribronchial tissue (Figure 1O). A few hours after the infection of the PCLS, a color change in the medium was observed, mainly for the PCLS infected with the moderately virulent strain 8067 and non-virulent strain T15. Therefore, the pH of the medium was measured. The most rapid change in medium pH was observed for PCLS infected with strain 8067, with acidification being evident by 6 hpi (Figure S2). For T15-infected PCLS, the acidification of the medium could be observed at 24 hpi (Figure S2). The pH of the medium from PCLS infected with S10 and uninfected controls remained similar throughout the experiment (Figure S2).

### 3.2. Transcriptional Host Response to S. suis Infection in the PCLS Model

**Figure 2:**
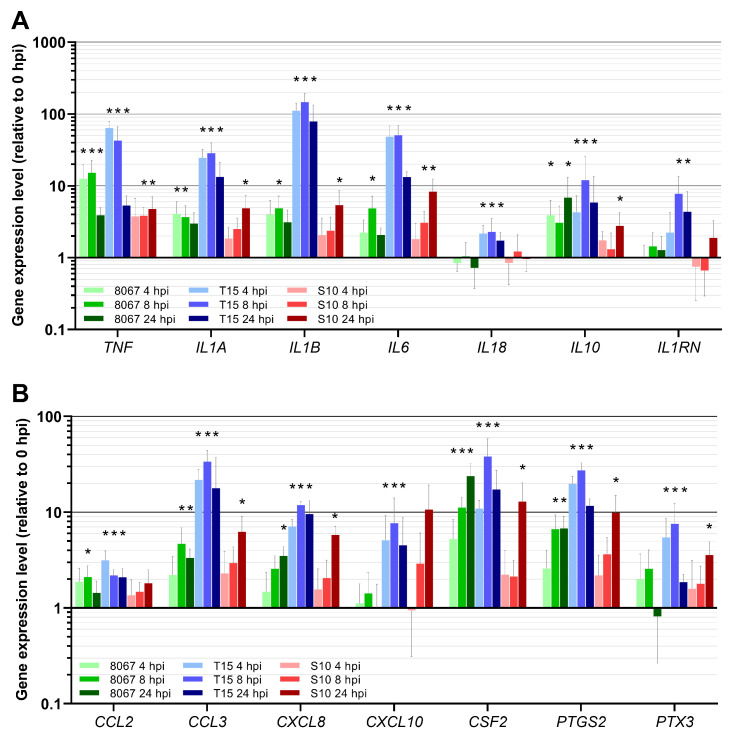
Porcine gene expression in PCLS infected with *S. suis* strain 8067, T15, or S10. (**A**) Genes coding for pro- and anti-inflammatory cytokines. (**B**) Chemokines and other inflammation-related protein coding genes. Expression levels at 4, 8, and 24 hpi have been scaled relative to expression levels at 0 hpi. Error bars depict 95% CI. * indicates statistically different expression levels relative to 0 hpi (Student’s *t* test, *p* < 0.05, FDR corrected). ** indicates the fold change is statistically significant (*p* < 0.05) at all two investigated time points. *** indicates the fold change is statistically significant (*p* < 0.05) at all three investigated time points.

The expression of 49 porcine genes was assayed in the ex vivo PCLS model during *S. suis* infection. The relative expression of porcine genes was quantified at 0, 4, 8, and 24 hpi in strain 8067-, T15-, S10-, and mock-infected PCLS samples (Figure 2). All porcine transcriptional results are summarized in Figure S3. No statistically significant changes in expression levels were detected for any of the assayed porcine genes in the mock-infected PCLS samples throughout the experiment (Figure S3A). A difference in the temporal dynamics of the host response to the moderately virulent strain 8067 and non-virulent strain T15 compared to the highly virulent S10 was observed for genes related to the inflammatory response, i.e., pro- and anti-inflammatory cytokines, chemokines, and other inflammation markers (Figure 2). For the 8067 and T15 strains, the general trend for these genes was that expression had already peaked at 4 or 8 hpi and was decreasing by 24 hpi. In contrast, during S10 infection, porcine gene expression consistently peaked at 24 hpi. Additionally, a significant increase in E-selectin (*SELE*) gene expression was observed only during 8067 and T15 infection, not during S10 infection (Figure S3B–D). PCA of genes related to the inflammatory response showed that PCLS infected with the three *S. suis* strains cluster distinctly from each other according to *S. suis* strain, with a slight overlap in PCLS infected with T15 and S10 (Figure S4A). No increase was observed in the transcription of Toll-like receptors (*TLR2*, *TLR4*, *TLR6*) and TLR adaptor protein *MYD88* during infection with any of the examined *S. suis* strains (Figure S3B–D). PCA of genes related to PRRs and transcription factors showed that PCLS infected with 8067 and T15 cluster together, distinct from PCLS infected with S10 (Figure S4B). Tight junction (TJ)-associated genes (*CLDN1*, *OCLN*) were not affected by *S. suis* infection in the PCLS model (Figure S3B–D). The pattern of expression of airway surface liquid (ASL) homeostasis-associated genes (*CFTR*, *BPIFA1*, *MUC1*, *MUC5AC*, *MUC5B*) in the PCLS varied greatly between the three strains, and only infection with strain T15 induced statistically significant changes in this group of genes (Figure S3B–D).

### 3.3. ELISA Quantification of Pro-Inflammatory Cytokines

**Figure 3:**
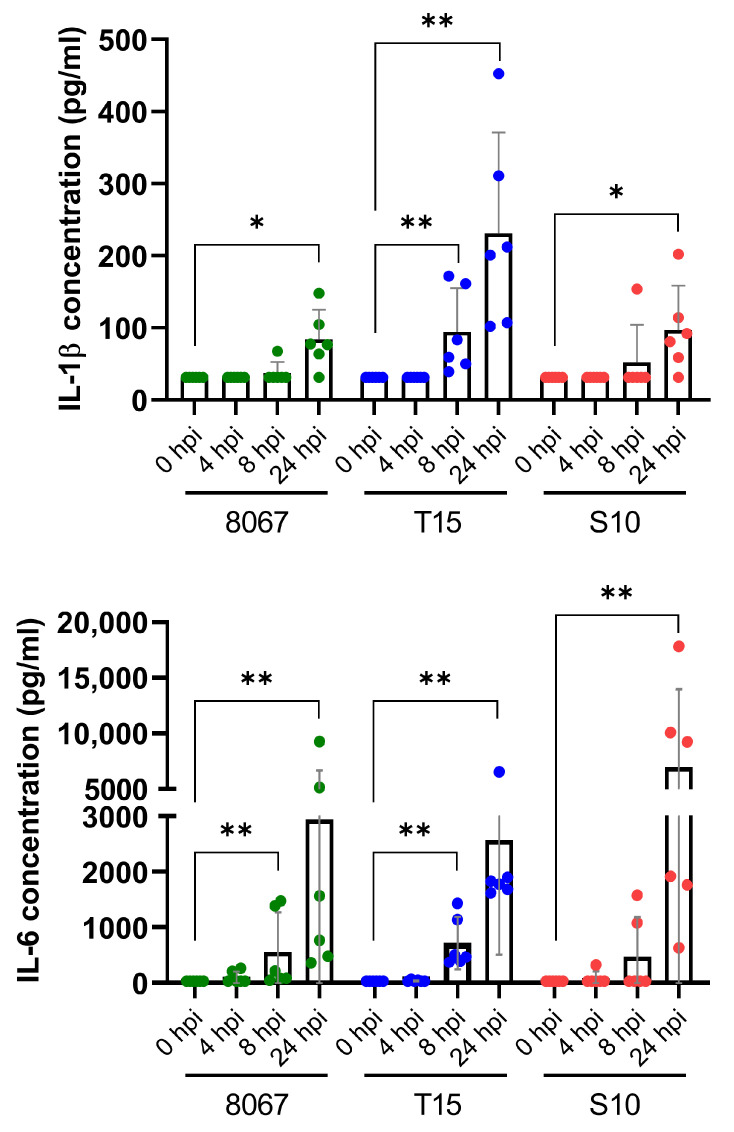
Quantification of pro-inflammatory cytokines in PCLS supernatants after *S. suis* infection (ELISA). Upper part: IL-1β. Lower part: IL-6. Error bars depict 95% CI. * *p* < 0.05; ** *p* < 0.005 (Mann–Whitney U test).

Transcriptional results were validated at the protein level for IL-1β and IL-6 (Figure 3). IL-6 concentrations were already significantly elevated in the supernatants at 8 hpi during infection with the moderately virulent strain 8067 and non-virulent strain T15 and remained elevated at 24 hpi. In contrast, IL-6 was only significantly elevated in supernatants of highly virulent strain S10-infected PCLS at 24 hpi. Similar to the IL-6 results, IL-1β was significantly elevated at 8 and 24 hpi during strain T15 infection, but only at 24 hpi during infection with strains 8067 and 10.

### 3.4. S. suis Gene Expression in the PCLS Model

**Figure 4:**
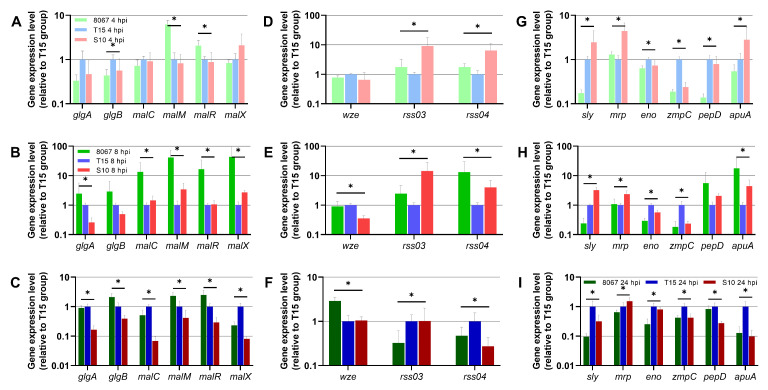
Relative expression levels of bacterial genes in *S. suis*-infected PCLS at 4 hpi (**A**,**D**,**G**), 8 hpi (**B**,**E**,**H**), and 24 hpi (**C**,**F**,**I**). In all graphs, expression levels of the T15 group have been scaled to 1, and expression levels of the 8067 and S10 groups have been scaled relative to the T15 group. Error bars depict 95% CI. * indicates statistically significant expression among the three *S. suis* strains for that gene (*p* < 0.05 (FDR corrected), one-way ANOVA).

The expression of 47 bacterial genes was assayed in the ex vivo PCLS model during *S. suis* infection. The relative expression of bacterial genes was quantified in strain 8067-, T15-, and S10-infected PCLS samples (Figure 4). All bacterial transcriptional results are summarized in Figure S5. The different strains used in this study impacted the outcome of host response as seen by the varied induction of genes involved in the innate immune response. In order to elucidate the differences observed in innate immunity, we simultaneously investigated the expression of selected bacterial virulence-associated and metabolic genes. Transcriptional analysis showed differential expression of bacterial genes between the three *S. suis* strains throughout the experiment (from 4 to 24 hpi) (Figure 4 and Figure S5). At all time points, the highly virulent S10 strain showed lower expression of metabolism-related genes (*glgA*, *glgB*, *malC*, *malM*, *malR*, *malX*) compared to the moderately virulent strain 8067 and/or the non-virulent strain T15, with only few statistically insignificant exceptions (Figure 4A–C). This correlated well with the pH decrease in the medium containing PCLSs infected with strains 8067 and T15 (Figure S2). At all time points, expression of the canonical *S. suis* virulence-associated cytolysin *sly* was lowest in strain 8067 compared to S10 and T15 (Figure 3G–I). The *sly* expression was higher in the S10 strain at 4 and 8 hpi compared to T15, but at 24 hpi, *sly* expression was higher in strain T15 than in either of strains S10 and 8067. The genes *mrp* and *pgdA* were highly expressed in S10 throughout the experiment compared to strains T15 and 8067 (Figure 3G–I and Figure S5). In addition, the small RNAs *rss03* and *rss04* were highly expressed at 4 hpi in S10 compared to T15 and 8067 (Figure 3D–F). For *zmpC* and *eno*, the expression was higher in strain T15 than in either of the strains S10 and 8067 throughout the experiment (Figure 3G–I). In general, higher expression of virulence-associated genes was observed at 4 hpi in S10 compared to T15 and 8067, whereas higher expression of metabolic genes was observed at 8 and 24 hpi in strain 8067 compared to S10 and T15. At 24 hpi, the highest expression of the majority of the genes was mainly seen in strain T15. PCA showed clear clustering of PCLS samples according to infecting *S. suis* strains at all individual time points (Figure S6). PCA of PCLS samples infected with strain 8067 showed that samples harvested at each time point clustered distinctly from each other (Figure S7A), whereas for strains T15 and S10, distinct clustering was seen for 24 hpi samples and the clustering of 4 and 8 hpi samples overlapped (Figure S7B–C).

## 4. Discussion

Previously, a number of virulence or virulence-associated factors have been identified in *Streptococcus suis*, as reviewed in [5]. *S. suis* is a genomically highly diverse pathobiont which resides in the tonsils of healthy pigs [1]. Despite the identification of various virulence-associated factors, there are a number of contradictions and controversies concerning these, which could be metabolic genes, surface proteins, or secreted proteins [5]. Precision-cut lung slices (PCLS) provide a representative 3D organ model, which mimics the in vivo lung architecture and enables simultaneous analysis of host and pathogen interplay in the lung [27]. In the present study, we investigated the transcriptional host response in a porcine PCLS model during infection with three *S. suis* strains of varying virulence potential: the highly virulent serotype 2 strain S10, the non-virulent serotype 2 strain T15, and the moderately virulent serotype 9 strain 8067. Despite the fact that there are limitations of PCLS, such as lack of cellular infiltration and adaptive immunity [41], the model proved to be a suitable tool for investigation of the pathogenic effect of *S. suis* in the porcine lung, evident from the observed induction of a host innate immune response mirroring findings from in vivo experimental challenge studies, including the transcriptional upregulation of *IL1B*, *IL6*, *IL18*, *TNF*, *CXCL8*, *SAA*, *SELE*, and *NFKBIA* [17,23].

An early peak in inflammation-related gene expression was detected at 4 or 8 hpi during infection with the non-virulent T15 strain as well as the moderately virulent 8067 strain, suggesting that the host is able to rapidly respond to the infection and control the inflammatory response induced by both these strains despite the difference in virulence potential. Evidence of this level of control was, however, not observed during highly virulent S10 infection: by the end of the experiment at 24 hpi, expression of inflammation-related genes was still on the rise. This mirrors results from a recent report of the porcine host response during in vivo experimental respiratory colonization with *S. suis* strains T15 and S10 [17]. It is likewise in accordance with the observed significant increase in *SELE* (E-selectin) expression during 8067 and T15 but not S10 infection, suggesting that the recruitment of immune cells to control the infection is initiated rapidly during 8067 and T15 infection. The decrease in expression of inflammation-related genes by 24 hpi in 8067- and T15-infected PCLS lends support to this notion. As such, the virulence observed during 8067 in vivo infection cannot be explained exclusively by the inflammation the strain induces, as the pattern of this response appears comparable to that of non-virulent T15 infection rather than highly virulent S10 infection. To this end, it is noteworthy that *PTX3* (pentraxin-3) is significantly upregulated during infection with both serotype 2 strains but not during 8067 infection. Pentraxins act as PRRs and acute phase proteins during infection and aid in the clearing of invading microorganisms by activating the complement system [42]. Porcine pentraxin-3 has likewise been shown to have antibacterial effects against *S. suis* (serotype 2) in vivo in piglets and mice [43].The absence of transcriptional induction of *PTX3* during 8067 infection might lead to impaired complement-dependent clearing of the bacterium compared to during S10 and T15 infection. This potentially impeded bacterial clearance could offer some explanation for the observed in vivo virulence of the 8067 strain, despite the limited inflammatory response that it induces in the PCLS model. It is also worth noting that only infection with the non-virulent strain T15 induced transcriptional upregulation of the pro-inflammatory cytokine *IL18*. Studies in mice have suggested a protective role for IL-18 during group B Streptococcus infection [44]. Furthermore, it has been shown that IL-18 is necessary for the induction of streptococcal toxic shock-like syndrome during infection with the virulent serotype 2 *S. suis* strain SC-19 [45].

Improved control of inflammation during 8067 and T15 infection compared to S10 infection is also evident in the significant increase in anti-inflammatory *IL10* expression already at 4 hpi; this is not achieved until 24 hpi during S10 infection. In addition, expression of the anti-inflammatory IL-1 receptor antagonist (IL-1RA), *IL1RN*, is significantly increased during non-virulent T15 infection. Perhaps this offers a partial explanation for the different clinical outcomes during in vivo challenge with the three strains; during T15 infection, IL-1RA is able to inhibit the pro-inflammatory effects induced by IL-1α and IL-1β, but this effect may be absent or impaired in 8067 and S10 infection due to the lack of increased *IL1RN* expression.

Prostaglandin endoperoxide synthase 2 (*PTGS2*), the gene encoding cyclooxygenase-2 (COX-2), was highly expressed at all time points in non-virulent T15-infected PCLSs, at 8 and 24 hpi in moderately virulent strain 8067-infected PCLS, and only at 24 hpi in highly virulent S10-infected PCLS. In a recent study by Dresen et al. [39], it was reported that *S. suis* induces COX-2 in a time-dependent manner in porcine PCLS. The highest induction of *PTGS2* in T15-infected PCLS in our study might be attributed to the higher expression of inflammatory cytokines such as *IL1B* and *TNF* in these samples.

In the PCLS model, we found no significant increase in the expression of PRRs in response to any of the three tested *S. suis* strains. Rather, the downregulation of *TLR4* and *TLR6* in response to T15 and S10 infection, respectively, as well as the downregulation of expression of the adapter protein *MYD88* during 8067 and T15 infection, was seen (albeit at low-fold changes, especially during T15 infection). The importance of MYD88-mediated signaling in the generation of an inflammatory response during *S. suis* infection has been demonstrated in vitro (murine dendritic cells, *S. suis* serotypes 2 and 9) [46,47,48] and in vivo (mouse model, *S. suis* serotype 2) [49,50], showing an almost entirely abolished cytokine response in the absence of *MYD88*. Similarly, recognition of *S. suis* by TLR2 contributes to this process, demonstrated by the somewhat impaired inflammatory response in the absence of *TLR2* [46,47,48,49,50]. Given the reported crucial importance of MYD88 for the inflammatory response, the observed downregulation of *MYD88* in the PCLS model could be another mechanism the host employs to hinder an exacerbated inflammatory response during 8067 and T15 infection, contributing to the observed lowered/lack of virulence of these strains in vivo compared to S10.

In a study of the genomic diversity of *S. suis* strains using comparative genomic hybridization (CGH), T15 was found to cluster more closely with 8067 than S10 [4], despite T15 and S10 being of the same serotype. This grouping is mirrored in our PCA of a selected group of genes representing the very early events during the host’s response to infection, namely genes related to bacterial recognition as well as transcription factors involved in the induction of the innate host response. This indicates that the host tissue is responding very differently to infection with S10 right from the beginning; the lower virulence of 8067 compared to S10 may, therefore, also in part be explained by its greater similarity to the host reaction pattern of bacterial recognition and downstream induction of transcription factors induced by the non-virulent T15, which might be governed by the greater genomic similarity found between strains 8067 and T15 [4]. 

For better understanding of the observed differences in innate immune response following the infection of porcine PCLS by the three *S. suis* strains, bacterial gene expression was investigated. Accordingly, genes involved in carbohydrate transport and metabolism were highly expressed in PCLS infected with strains 8067 and T15. In line with this, more acidification of culture medium was observed in PCLS infected with strain 8067 and T15, reflecting the higher metabolic activity of these strains. Little or no acidification was observed in the culture medium of PCLS infected with S10, even at 24 hpi. The number of bacteria during the experimental period remained comparable between the strains, despite the difference in the acidification of the media. With this observation, we hypothesize that the higher metabolism of the strains 8067 and T15 lead to a faster recognition by the innate immune response, which can be further inferred to the observation in vivo. Moreover, virulence-associated genes, such as *sly*, *mrp*, and *pgdA*, were highly expressed in PCLS infected with S10. 

In conclusion, the current study demonstrated the strain-dependent induction of genes involved in the innate immune response, reflecting the virulence potential among the investigated strains. This was evident from the highest induction of gene coding for pro- and anti-inflammatory cytokines in porcine PCLS infected with the non-virulent strain T15 and moderately virulent strain 8067 at the early stages of infection compared to the delayed response in highly virulent S10 infection. The analysis of bacterial gene expression in the infected PCLS revealed higher induction of bacterial genes involved in carbohydrate transport and metabolism in PCLS infected with strains 8067 and T15 compared to those infected with S10. We suggest that the higher metabolic activity of these strains could lead to faster recognition by the host innate immune response. Previously identified virulence-associated factors were highly expressed in PCLS infected with S10, which supports the validity of the PCLS model for investigation of the virulence potential of *S. suis* strains in the lung. Further studies need to be conducted to prove the individual factors leading to these differences.

## Data Availability

Relative expression levels for all porcine and bacterial genes quantifiable in the PCLS model are available in Supplementary Table S1. All ELISA results on IL-1β and IL-6 concentrations in PCLS supernatants are available in Supplementary Table S2.

## Supplementary Materials

The following supporting information can be downloaded at: https://www.mdpi.com/article/10.3390/pathogens13010004/s1. Figure S1. Growth curves for *Streptococcus suis* strains 8067, S10, and T15 in culture supernatants of porcine PCLSs; Figure S2. pH of supernatants from PCLS: uninfected controls and infected with *S. suis* strains 8067, T15, and S10. (A) Acidification of the medium in slices infected with 8067 were clearly visible after 6 hpi and for T15 at 24 hpi. pH of the medium remained comparable between slices infected with S10, uninfected controls, and medium only. (B) pH of supernatants from uninfected controls and slices infected with the three *S. suis* strains tested by another pH indicator; Figure S3. Transcriptional levels of porcine genes in mock- and *S. suis*-infected PCLS. Error bars depict 95% CI. * indicates statistically different expression levels relative to 0 hpi (Student’s *t* test, *p* < 0.05, FDR corrected); Figure S4. PCA of porcine gene expression. (A) Genes related to inflammation: includes genes *IL1A*, *IL1B*, *IL6*, *IL18*, *TNF*, *CCL2*, *CCL3*, *CXCL8*, *CXCL10*, *CSF2*, *PTGS2*, *IL10*, *IL1RN*, *SAA*, *PTX3*. (B) Genes related to PRRs and transcription factors: includes genes *TLR2*, *TLR4*, *TLR6*, *MYD88*, *NFKB1*, *NFKBIA*, *IRF1*, *JUN*. Green—8067. Blue—T15. Red—S10. Squares—4 hpi. Circles—8 hpi. Triangles—24 hpi; Figure S5. Bacterial gene expression in PCLS. (A) Strain 8067. (B) Strain T15. (C) Strain S10. Expression levels at 4, 8, and 24 hpi have been scaled relative to expression levels at 0 hpi. Error bars depict 95% CI. * indicates statistically significant expression among the three *S. suis* strains for that gene (*p* < 0.05 (FDR corrected), one-way ANOVA); Figure S6. PCA of bacterial gene expression in porcine PCLS from 4 to 24 hpi. PCA is based on the expression levels of all genes that were successfully quantified in all three *S. suis* strains. (A) 4 hpi. (B) 8 hpi. (C) 24 hpi. Green—strain 8067. Blue—strain T15. Red—strain S10; Figure S7. PCA of *S. suis* strains 8067, T15, and S10 gene expression in porcine PCLS. PCA is based on the expression levels of all genes that were successfully quantified in all three *S. suis* strains. (A) Strain 8067. (B) Strain T15. (C) Strain S10. White—4 hpi. Grey—8 hpi. Black—24 hpi; Supplementary Table S1. Relative expression levels for all porcine and bacterial genes quantifiable in the PCLS model. Supplementary Table S2. All ELISA results on IL-1β and IL-6 concentrations in PCLS supernatants.

## References

[1] Votsch D., Willenborg M., Weldearegay Y.B., Valentin-Weigand P. (2018). *Streptococcus suis*—The “Two Faces” of a Pathobiont in the Porcine Respiratory Tract. Front. Microbiol..

[2] Haas B., Grenier D. (2018). Understanding the virulence of *Streptococcus suis*: A veterinary, medical, and economic challenge. Med. Et Mal. Infect..

[3] Hlebowicz M., Jakubowski P., Smiatacz T. (2019). *Streptococcus suis* Meningitis: Epidemiology, Clinical Presentation and Treatment. Vector Borne Zoonotic Dis..

[4] de Greeff A., Wisselink H.J., de Bree F.M., Schultsz C., Baums C.G., Thi H.N., Stockhofe-Zurwieden N., Smith H.E. (2011). Genetic diversity of *Streptococcus suis* isolates as determined by comparative genome hybridization. BMC Microbiol..

[5] Segura M., Fittipaldi N., Calzas C., Gottschalk M. (2017). Critical *Streptococcus suis* Virulence Factors: Are They All Really Critical?. Trends Microbiol..

[6] Fittipaldi N., Segura M., Grenier D., Gottschalk M. (2012). Virulence factors involved in the pathogenesis of the infection caused by the swine pathogen and zoonotic agent *Streptococcus suis*. Future Microbiol..

[7] Baums C.G., Valentin-Weigand P. (2009). Surface-associated and secreted factors of *Streptococcus suis* in epidemiology, pathogenesis and vaccine development. Anim. Health Res. Rev..

[8] Silva L.M., Baums C.G., Rehm T., Wisselink H.J., Goethe R., Valentin-Weigand P. (2006). Virulence-associated gene profiling of *Streptococcus suis* isolates by PCR. Vet. Microbiol..

[9] Lecours M.P., Gottschalk M., Houde M., Lemire P., Fittipaldi N., Segura M. (2011). Critical role for *Streptococcus suis* cell wall modifications and suilysin in resistance to complement-dependent killing by dendritic cells. J. Infect. Dis..

[10] Li G., Wang G., Si X., Zhang X., Liu W., Li L., Wang J. (2019). Inhibition of suilysin activity and inflammation by myricetin attenuates *Streptococcus suis* virulence. Life Sci..

[11] Tenenbaum T., Asmat T., Seitz M., Schroten H., Schwerk C. (2016). Biological activities of suilysin: Role in *Streptococcus suis* pathogenesis. Future Microbiol..

[12] Tharavichitkul P., Wongsawan K., Takenami N., Pruksakorn S., Fongcom A., Gottschalk M., Khanthawa B., Supajatura V., Takai S. (2014). Correlation between PFGE Groups and mrp/epf/sly Genotypes of Human *Streptococcus suis* Serotype 2 in Northern Thailand. J. Pathog..

[13] Jacobs A.A., Loeffen P.L., van den Berg A.J., Storm P.K. (1994). Identification, purification, and characterization of a thiol-activated hemolysin (suilysin) of *Streptococcus suis*. Infect. Immun..

[14] Roy D., Auger J.P., Segura M., Fittipaldi N., Takamatsu D., Okura M., Gottschalk M. (2015). Role of the capsular polysaccharide as a virulence factor for *Streptococcus suis* serotype 14. Can. J. Vet. Res..

[15] Segura M., Gottschalk M., Olivier M. (2004). Encapsulated *Streptococcus suis* inhibits activation of signaling pathways involved in phagocytosis. Infect. Immun..

[16] Smith H.E., Damman M., van der Velde J., Wagenaar F., Wisselink H.J., Stockhofe-Zurwieden N., Smits M.A. (1999). Identification and characterization of the cps locus of *Streptococcus suis* serotype 2: The capsule protects against phagocytosis and is an important virulence factor. Infect. Immun..

[17] Neila-Ibanez C., Brogaard L., Pailler-Garcia L., Martinez J., Segales J., Segura M., Heegaard P.M.H., Aragon V. (2021). Piglet innate immune response to *Streptococcus suis* colonization is modulated by the virulence of the strain. Vet. Res..

[18] Liu M., Tan C., Fang L., Xiao S., Chen H. (2011). Microarray analyses of THP-1 cells infected with *Streptococcus suis* serotype 2. Vet. Microbiol..

[19] Graveline R., Segura M., Radzioch D., Gottschalk M. (2007). TLR2-dependent recognition of *Streptococcus suis* is modulated by the presence of capsular polysaccharide which modifies macrophage responsiveness. Int. Immunol..

[20] Zheng H., Luo X., Segura M., Sun H., Ye C., Gottschalk M., Xu J. (2012). The role of toll-like receptors in the pathogenesis of *Streptococcus suis*. Vet. Microbiol..

[21] Meijerink M., Ferrando M.L., Lammers G., Taverne N., Smith H.E., Wells J.M. (2012). Immunomodulatory effects of *Streptococcus suis* capsule type on human dendritic cell responses, phagocytosis and intracellular survival. PLoS ONE.

[22] Dang Y., Lachance C., Wang Y., Gagnon C.A., Savard C., Segura M., Grenier D., Gottschalk M. (2014). Transcriptional approach to study porcine tracheal epithelial cells individually or dually infected with swine influenza virus and *Streptococcus suis*. BMC Vet. Res..

[23] Liu M., Fang L., Tan C., Long T., Chen H., Xiao S. (2011). Understanding *Streptococcus suis* serotype 2 infection in pigs through a transcriptional approach. BMC Genom..

[24] Russell W.M.S., Burch R.L. (1959). The Principles of Humane Experimental Technique.

[25] Meng F., Punyadarsaniya D., Uhlenbruck S., Hennig-Pauka I., Schwegmann-Wessels C., Ren X., Durrwald R., Herrler G. (2013). Replication characteristics of swine influenza viruses in precision-cut lung slices reflect the virulence properties of the viruses. Vet. Res..

[26] Weldearegay Y.B., Muller S., Hanske J., Schulze A., Kostka A., Ruger N., Hewicker-Trautwein M., Brehm R., Valentin-Weigand P., Kammerer R. (2019). Host-Pathogen Interactions of *Mycoplasma mycoides* in Caprine and Bovine Precision-Cut Lung Slices (PCLS) Models. Pathogens.

[27] Reamon-Buettner S.M., Niehof M., Hirth N., Danov O., Obernolte H., Braun A., Warnecke J., Sewald K., Wronski S. (2019). Transcriptomic Analysis Reveals Priming of The Host Antiviral Interferon Signaling Pathway by Bronchobini((R)) Resulting in Balanced Immune Response to Rhinovirus Infection in Mouse Lung Tissue Slices. Int. J. Mol. Sci..

[28] Henjakovic M., Martin C., Hoymann H.G., Sewald K., Ressmeyer A.R., Dassow C., Pohlmann G., Krug N., Uhlig S., Braun A. (2008). Ex vivo lung function measurements in precision-cut lung slices (PCLS) from chemical allergen-sensitized mice represent a suitable alternative to in vivo studies. Toxicol. Sci..

[29] Henjakovic M., Sewald K., Switalla S., Kaiser D., Muller M., Veres T.Z., Martin C., Uhlig S., Krug N., Braun A. (2008). Ex vivo testing of immune responses in precision-cut lung slices. Toxicol. Appl. Pharmacol..

[30] Lyons-Cohen M.R., Thomas S.Y., Cook D.N., Nakano H. (2017). Precision-cut Mouse Lung Slices to Visualize Live Pulmonary Dendritic Cells. J. Vis. Exp..

[31] Temann A., Golovina T., Neuhaus V., Thompson C., Chichester J.A., Braun A., Yusibov V. (2017). Evaluation of inflammatory and immune responses in long-term cultured human precision-cut lung slices. Hum. Vaccines Immunother..

[32] Brogaard L., Klitgaard K., Heegaard P.M., Hansen M.S., Jensen T.K., Skovgaard K. (2015). Concurrent host-pathogen gene expression in the lungs of pigs challenged with *Actinobacillus pleuropneumoniae*. BMC Genom..

[33] Vecht U., Arends J.P., van der Molen E.J., van Leengoed L.A. (1989). Differences in virulence between two strains of *Streptococcus suis* type II after experimentally induced infection of newborn germ-free pigs. Am. J. Vet. Res..

[34] Paddenberg R., Mermer P., Goldenberg A., Kummer W. (2014). Videomorphometric analysis of hypoxic pulmonary vasoconstriction of intra-pulmonary arteries using murine precision cut lung slices. J. Vis. Exp..

[35] Punyadarsaniya D., Liang C.H., Winter C., Petersen H., Rautenschlein S., Hennig-Pauka I., Schwegmann-Wessels C., Wu C.Y., Wong C.H., Herrler G. (2011). Infection of differentiated porcine airway epithelial cells by influenza virus: Differential susceptibility to infection by porcine and avian viruses. PLoS ONE.

[36] Niehof M., Hildebrandt T., Danov O., Arndt K., Koschmann J., Dahlmann F., Hansen T., Sewald K. (2017). RNA isolation from precision-cut lung slices (PCLS) from different species. BMC Res. Notes.

[37] Vandesompele J., De Preter K., Pattyn F., Poppe B., Van Roy N., De Paepe A., Speleman F. (2002). Accurate normalization of real-time quantitative RT-PCR data by geometric averaging of multiple internal control genes. Genome Biol..

[38] Andersen C.L., Jensen J.L., Orntoft T.F. (2004). Normalization of real-time quantitative reverse transcription-PCR data: A model-based variance estimation approach to identify genes suited for normalization, applied to bladder and colon cancer data sets. Cancer Res..

[39] Dresen M., Schenk J., Berhanu Weldearegay Y., Votsch D., Baumgartner W., Valentin-Weigand P., Nerlich A. (2021). *Streptococcus suis* Induces Expression of Cyclooxygenase-2 in Porcine Lung Tissue. Microorganisms.

[40] Votsch D., Willenborg M., Baumgartner W., Rohde M., Valentin-Weigand P. (2021). Bordetella bronchiseptica promotes adherence, colonization, and cytotoxicity of Streptococcus suis in a porcine precision-cut lung slice model. Virulence.

[41] Viana F., O’Kane C.M., Schroeder G.N. (2022). Precision-cut lung slices: A powerful ex vivo model to investigate respiratory infectious diseases. Mol. Microbiol..

[42] Haapasalo K., Meri S. (2019). Regulation of the Complement System by Pentraxins. Front. Immunol..

[43] Xu J., Mu Y., Zhang Y., Dong W., Zhu Y., Ma J., Song W., Pan Z., Lu C., Yao H. (2015). Antibacterial effect of porcine PTX3 against *Streptococcus suis* type 2 infection. Microb. Pathog..

[44] Mancuso G., Midiri A., Beninati C., Zummo S., Biondo C. (2021). Protective role of IL-18 in host defenses against group B *Streptococcus*. Eur. J. Clin. Microbiol. Infect. Dis..

[45] Xu L., Zeng Y., Gao P., Lu X., Xia K., Zhou L., Zhang C., Yi C., Zhang A. (2022). IL-18 Signaling Is Essential for Causing Streptococcal Toxic Shock-like Syndrome (STSLS). Life.

[46] Lecours M.P., Segura M., Fittipaldi N., Rivest S., Gottschalk M. (2012). Immune receptors involved in Streptococcus suis recognition by dendritic cells. PLoS ONE.

[47] Lavagna A., Auger J.P., Girardin S.E., Gisch N., Segura M., Gottschalk M. (2020). Recognition of Lipoproteins by Toll-like Receptor 2 and DNA by the AIM2 Inflammasome Is Responsible for Production of Interleukin-1beta by Virulent Suilysin-negative *Streptococcus suis* Serotype 2. Pathogens.

[48] Auger J.P., Boa A.C., Segura M., Gottschalk M. (2019). Antigen I/II Participates in the Interactions of *Streptococcus suis* Serotype 9 With Phagocytes and the Development of Systemic Disease. Front. Cell Infect. Microbiol..

[49] Auger J.P., Benoit-Biancamano M.O., Bedard C., Segura M., Gottschalk M. (2019). Differential role of MyD88 signaling in *Streptococcus suis* serotype 2-induced systemic and central nervous system diseases. Int. Immunol..

[50] Lavagna A., Auger J.P., Dumesnil A., Roy D., Girardin S.E., Gisch N., Segura M., Gottschalk M. (2019). Interleukin-1 signaling induced by *Streptococcus suis* serotype 2 is strain-dependent and contributes to bacterial clearance and inflammation during systemic disease in a mouse model of infection. Vet. Res..

