# Supplementary material for: Transcriptional Host Responses to Infection with Streptococcus suis in a Porcine Precision-Cut Lung Slice Model: Between-Strain Differences Suggest Association with Virulence Potential
Source: Pathogens. 2023 Dec 19;13(1):4. doi: 10.3390/pathogens13010004 (PMC10820225; doi:10.3390/pathogens13010004)

Figure S1. Growth curves for *Streptococcus suis* strains 8067, S10, and T15 in culture supernatants of porcine PCLS.

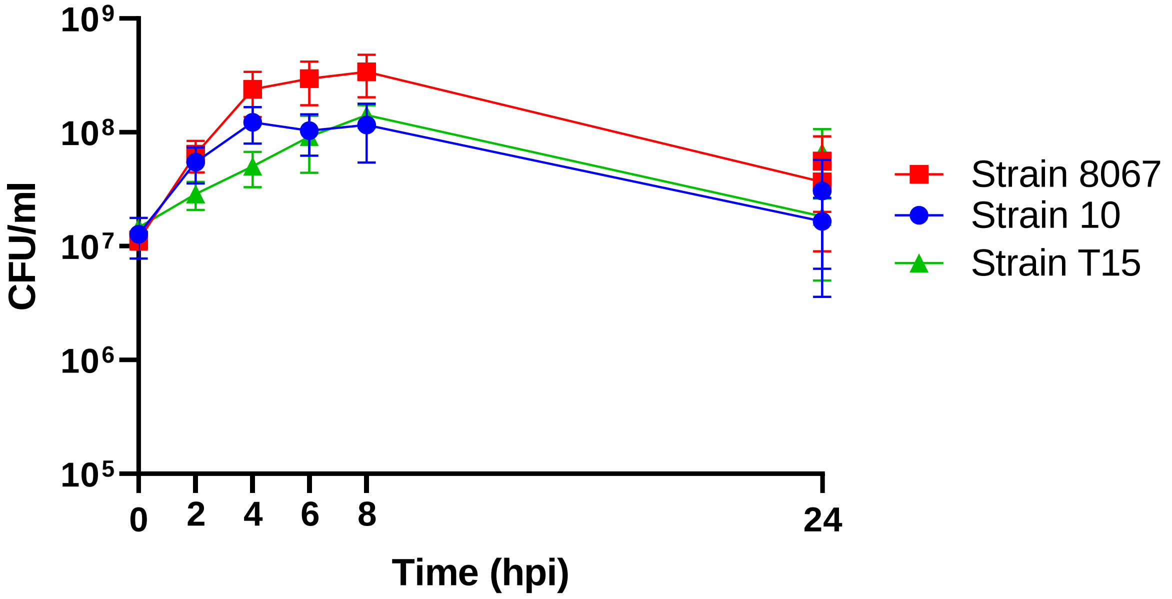

Figure S2. pH of supernatants from PCLS uninfected controls and infected with *S. suis* strains 8067, T15 and S10. A) Acidification of the medium in slices infected with 8067 were clearly visible after 6 hpi and for T15 at 24 hpi. pH of the medium remained comparable between slices infected with S10, uninfected controls and medium only. B) pH of supernatants from uninfected controls and slices infected with the three *S. suis* strains tested by another pH indicator.

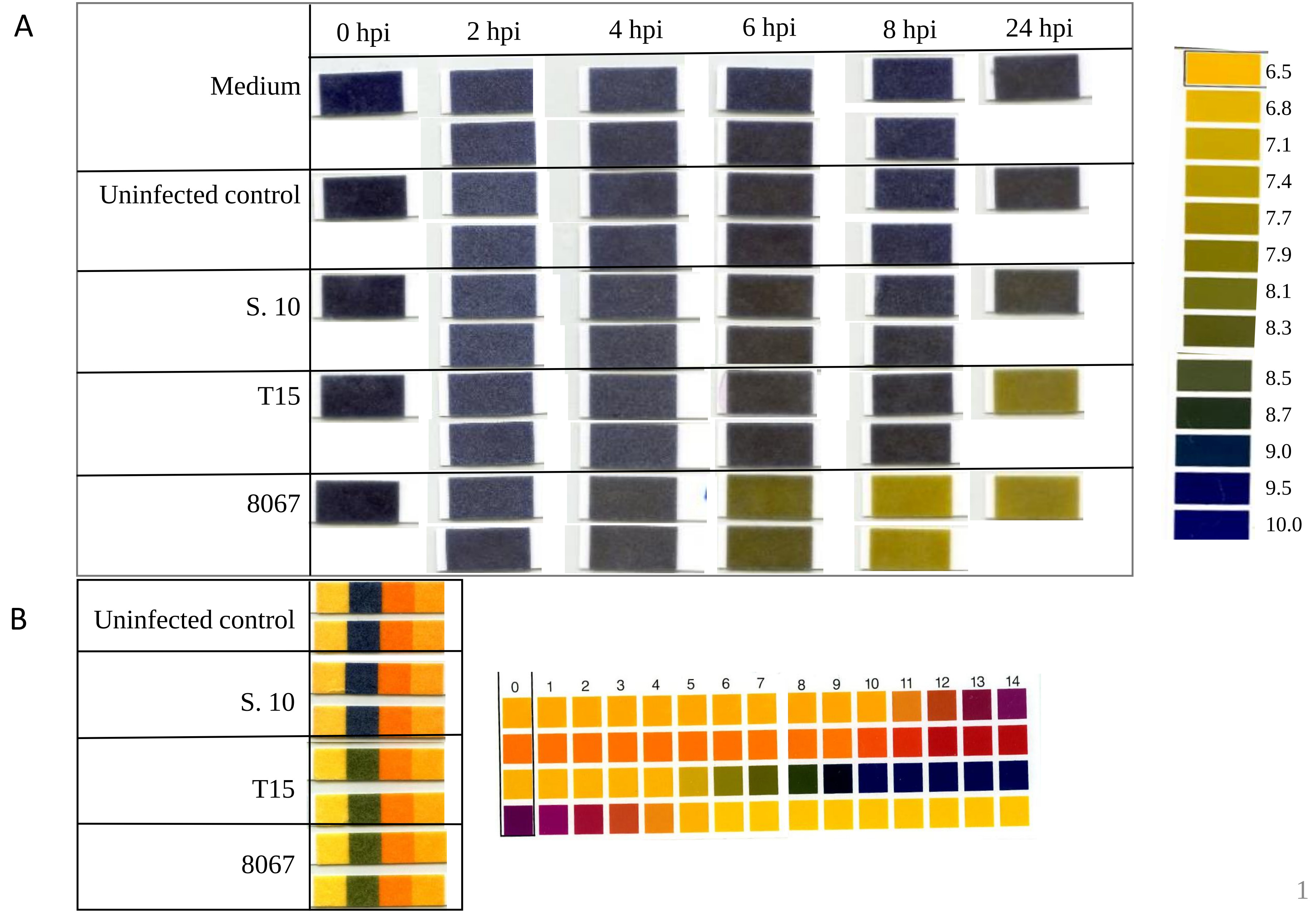

Figure S3. Porcine gene expression in PCLS. A) mock-infected PCLS. B) strain 8067 infected PCLS. C) strain T15 infected PCLS. D) strain S10 infected PCLS Expression levels at 4, 8, and 24 hpi have been scaled relative to expression levels at 0 hpi. Error bars depict 95 % CI. * indicates statistically different expression levels relative to 0 hpi (Student's *t* test, $p < 0.05$, FDR corrected).

A

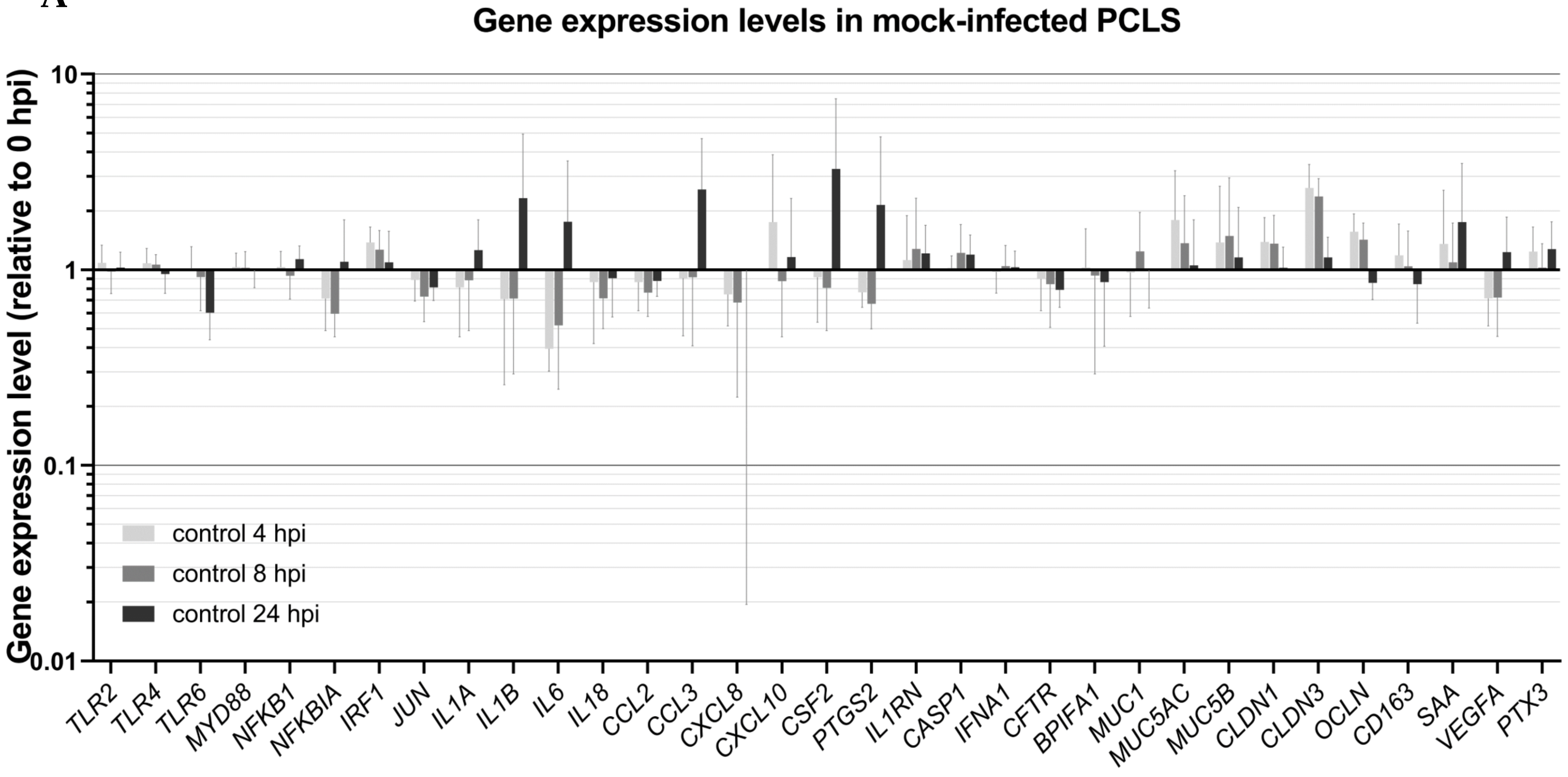

B

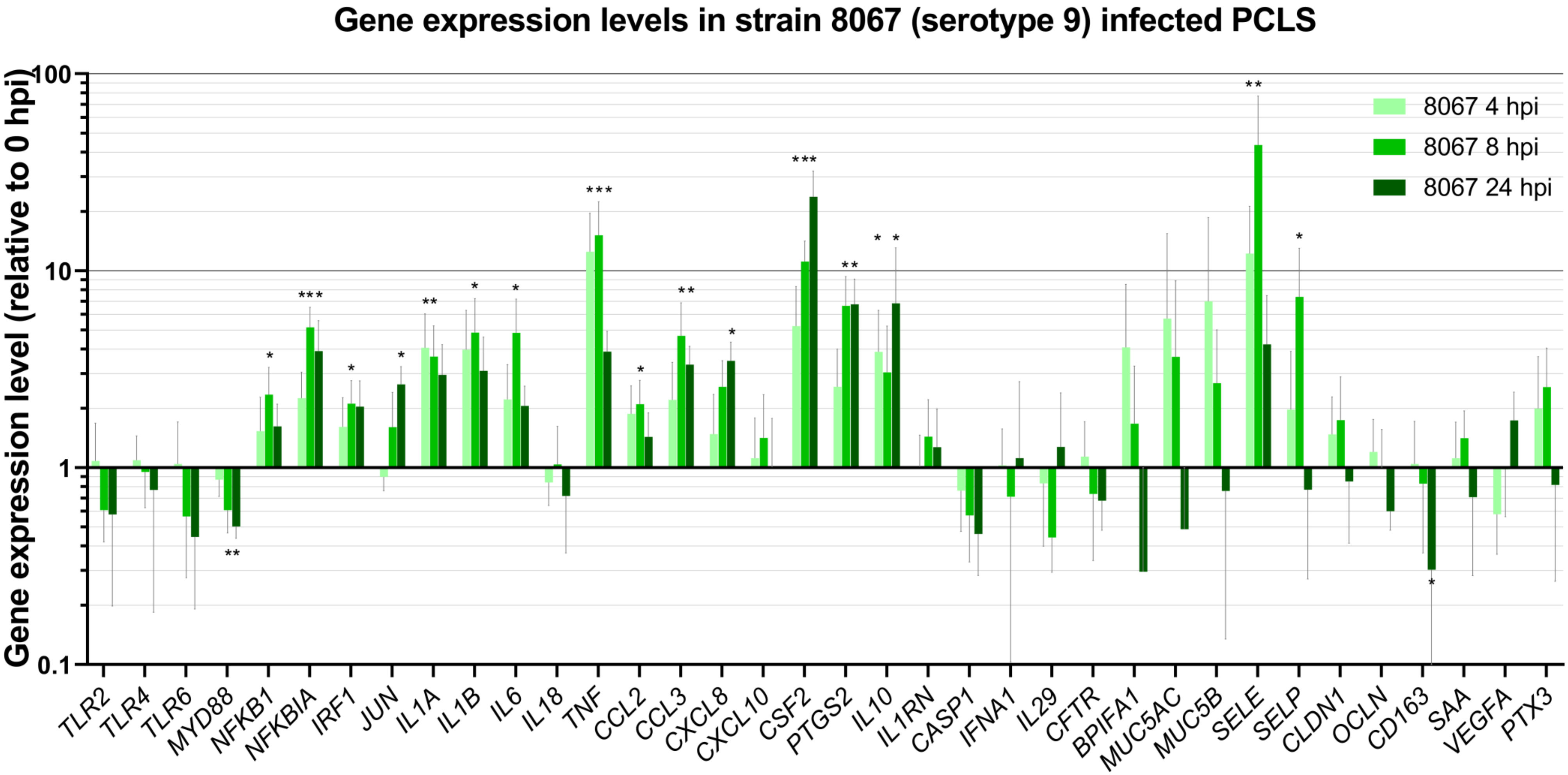

Gene expression levels in strain 8067 (serotype 9) infected PCLS
Gene expression level (relative to 0 hpi)
100
10
1
0.1
8067 4 hpi
8067 8 hpi
8067 24 hpi
TLR2
TLR4
TLR6
MYD88
NFKB1
NFKBIA
IRF1
JUN
IL1A
IL1B
IL6
IL18
TNF
CCL2
CCL3
CXCL8
CXCL10
CSF2
PTGS2
IL10
IL1RN
CASP1
IFNA1
IL29
CFTR
BPIFA1
MUC5AC
MUC5B
SELE
SELP
CLDN1
OCLN
CD163
SAA
VEGFA
PTX3

C

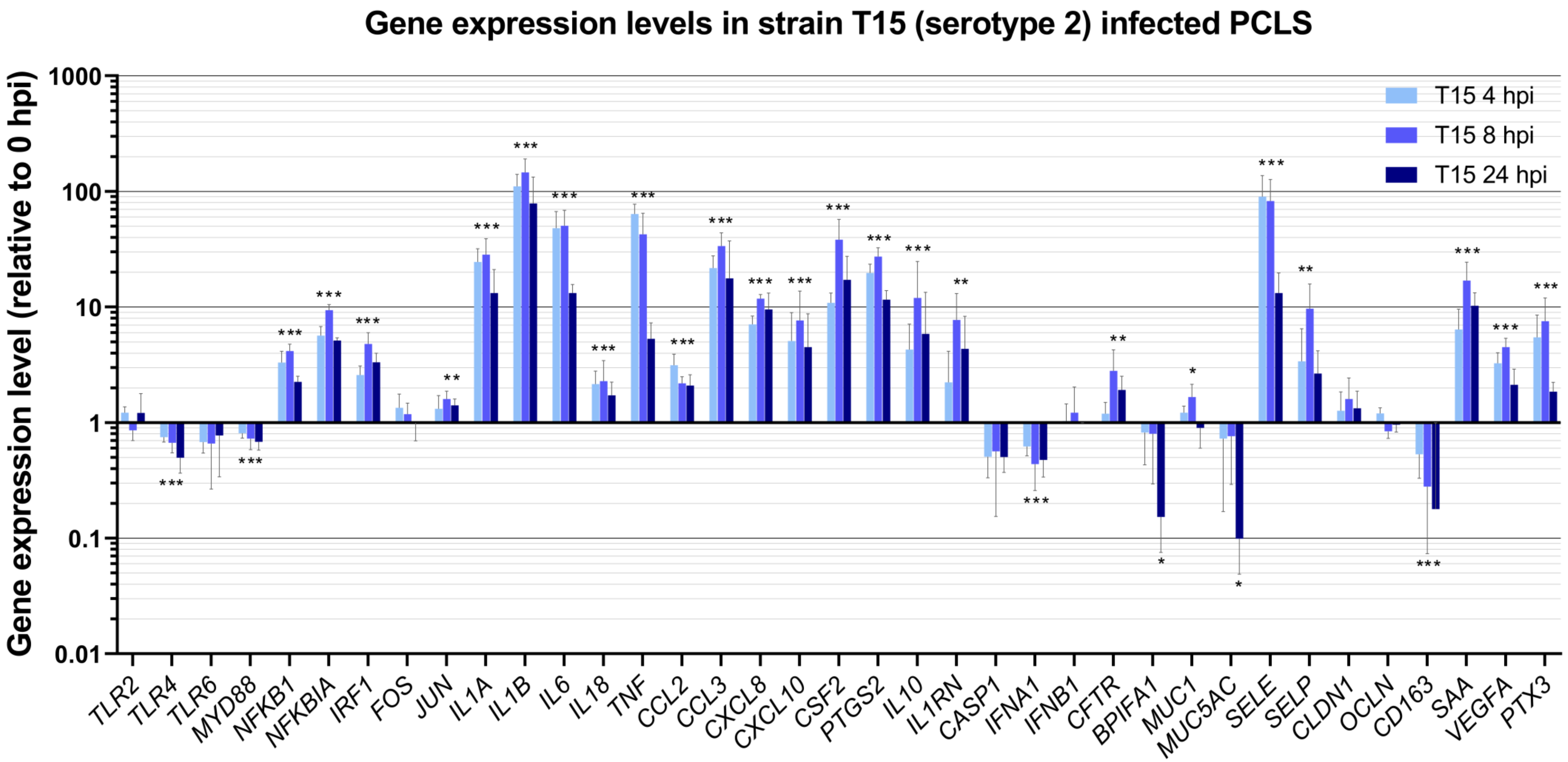

Gene expression levels in strain T15 (serotype 2) infected PCLS
Gene expression level (relative to 0 hpi)
1000
100
10
1
0.1
0.01
T15 4 hpi
T15 8 hpi
T15 24 hpi
TLR2
TLR4
TLR6
MYD88
NFKB1
NFKBIA
IRF1
FOS
JUN
IL1A
IL1B
IL6
IL18
TNF
CCL2
CCL3
CXCL8
CXCL10
CSF2
PTGS2
IL10
IL1RN
CASP1
IFNA1
IFNB1
CFTR
BPIFA1
MUC1
MUC5AC
SELE
SELP
CLDN1
OCLN
CD163
SAA
VEGFA
PTX3

D

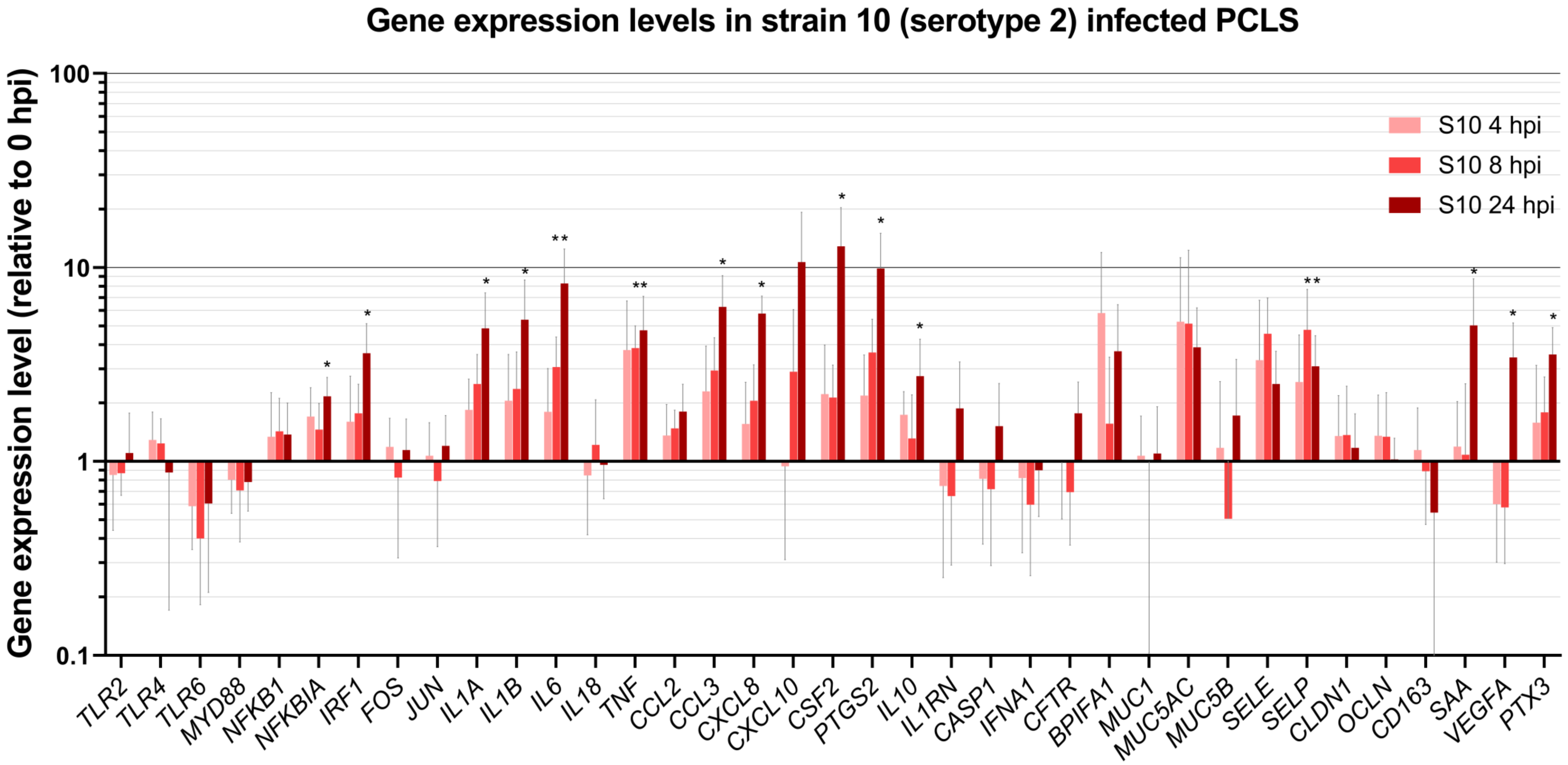
Gene expression levels in strain 10 (serotype 2) infected PCLS
Gene expression level (relative to 0 hpi)
100
10
1
0.1
S10 4 hpi
S10 8 hpi
S10 24 hpi
TLR2
TLR4
TLR6
MYD88
NFKB1
NFKBIA
IRF1
FOS
JUN
IL1A
IL1B
IL6
IL18
TNF
CCL2
CCL3
CXCL8
CXCL10
CSF2
PTGS2
IL10
IL1RN
CASP1
IFNA1
CFTR
BPIFA1
MUC1
MUC5AC
MUC5B
SELE
SELP
CLDN1
OCLN
CD163
SAA
VEGFA
PTX3

Figure S4. PCA of porcine gene expression. A) Genes related to inflammation; includes genes *IL1A*, *IL1B*, *IL6*, *IL18*, *TNF*, *CCL2*, *CCL3*, *CXCL8*, *CXCL10*, *CSF2*, *PTGS2*, *IL10*, *IL1RN*, *SAA*, *PTX3*. B) Genes related to PRRs and transcription factors, includes genes *TLR2*, *TLR4*, *TLR6*, *MYD88*, *NFKB1*, *NFKBIA*, *IRF1*, *JUN*. Green – 8067. Blue – T15. Red – S10. Squares – 4 hpi. Circles – 8 hpi. Triangles – 24 hpi.

A

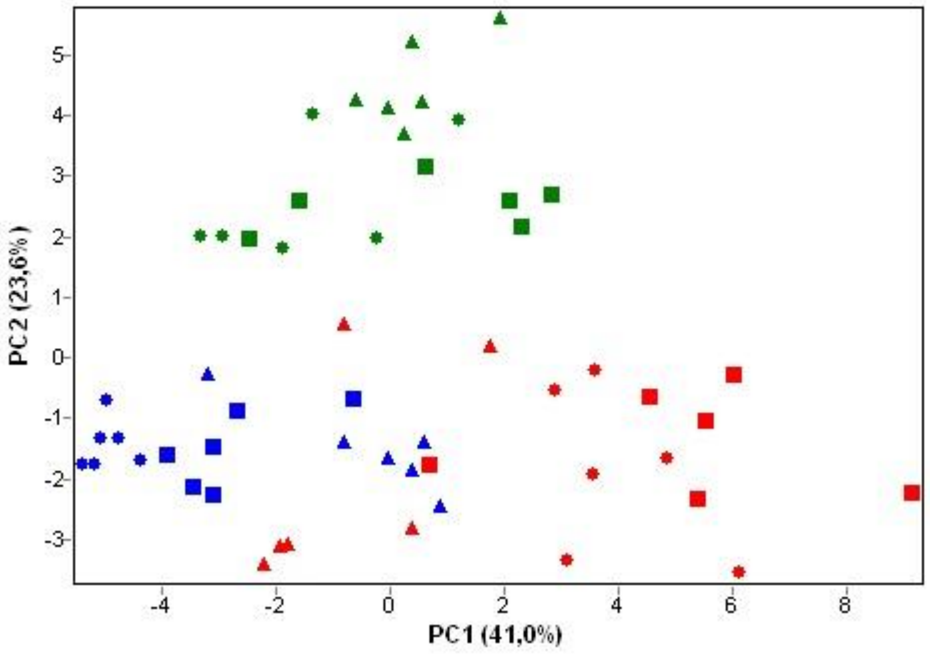

B

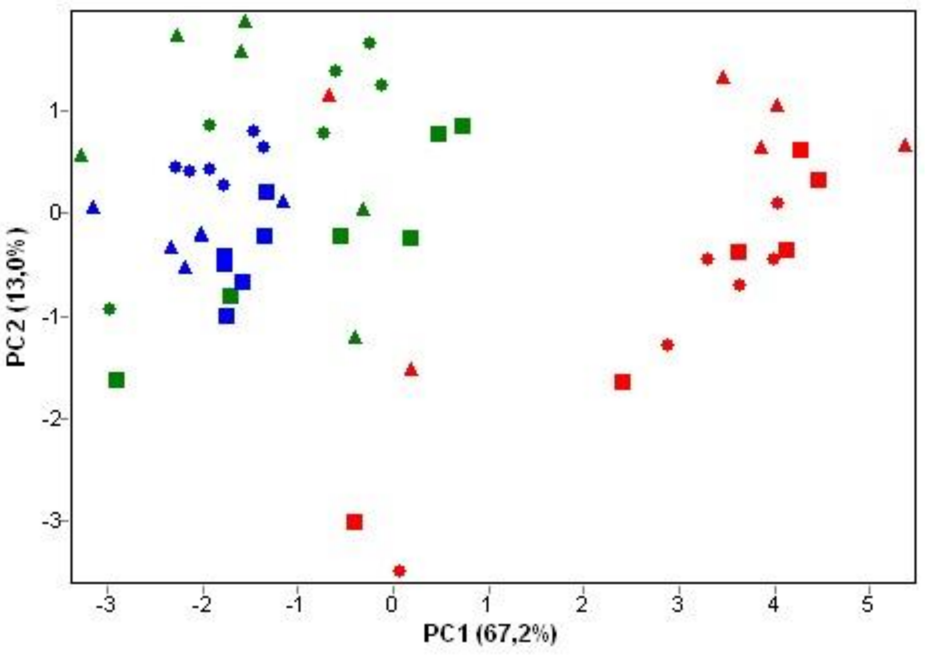

Figure S5. Bacterial gene expression in PCLS. A) strain 8067. B) strain T15. C) strain S10. Expression levels at 4, 8, and 24 hpi have been scaled relative to expression levels at 0 hpi. Error bars depict 95 % CI. * indicates statistically significant expression among the three *S. suis* strains for that gene ($p < 0.05$ (FDR corrected), one-way ANOVA).

A

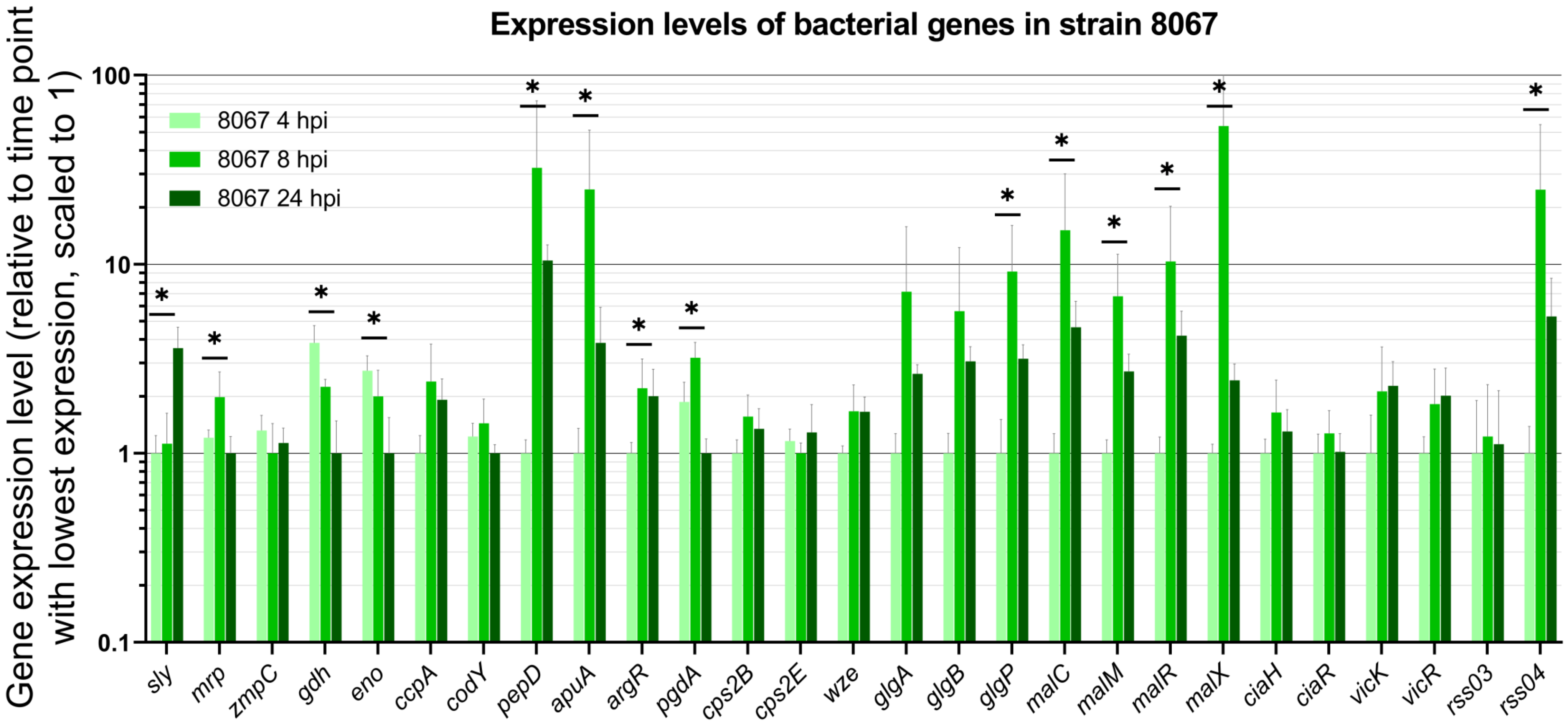

B

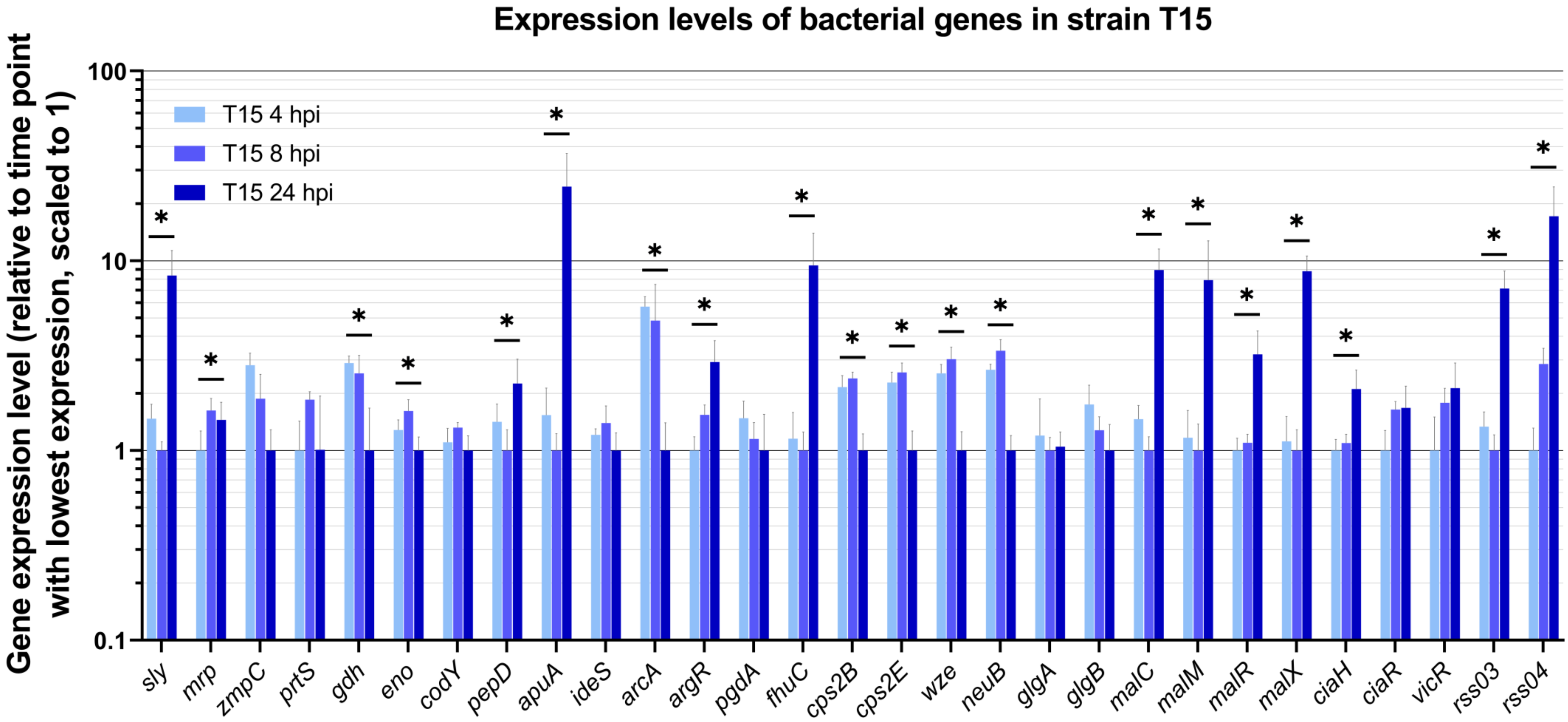

Expression levels of bacterial genes in strain T15
Gene expression level (relative to time point with lowest expression, scaled to 1)
100
10
1
0.1
T15 4 hpi
T15 8 hpi
T15 24 hpi
sly
mrp
zmpC
prtS
gdh
eno
codY
pepD
apuA
ideS
arcA
argR
pgdA
fhuC
cps2B
cps2E
wze
neuB
glgA
glgB
malC
malM
malR
malX
ciaH
ciaR
vicR
rss03
rss04

C

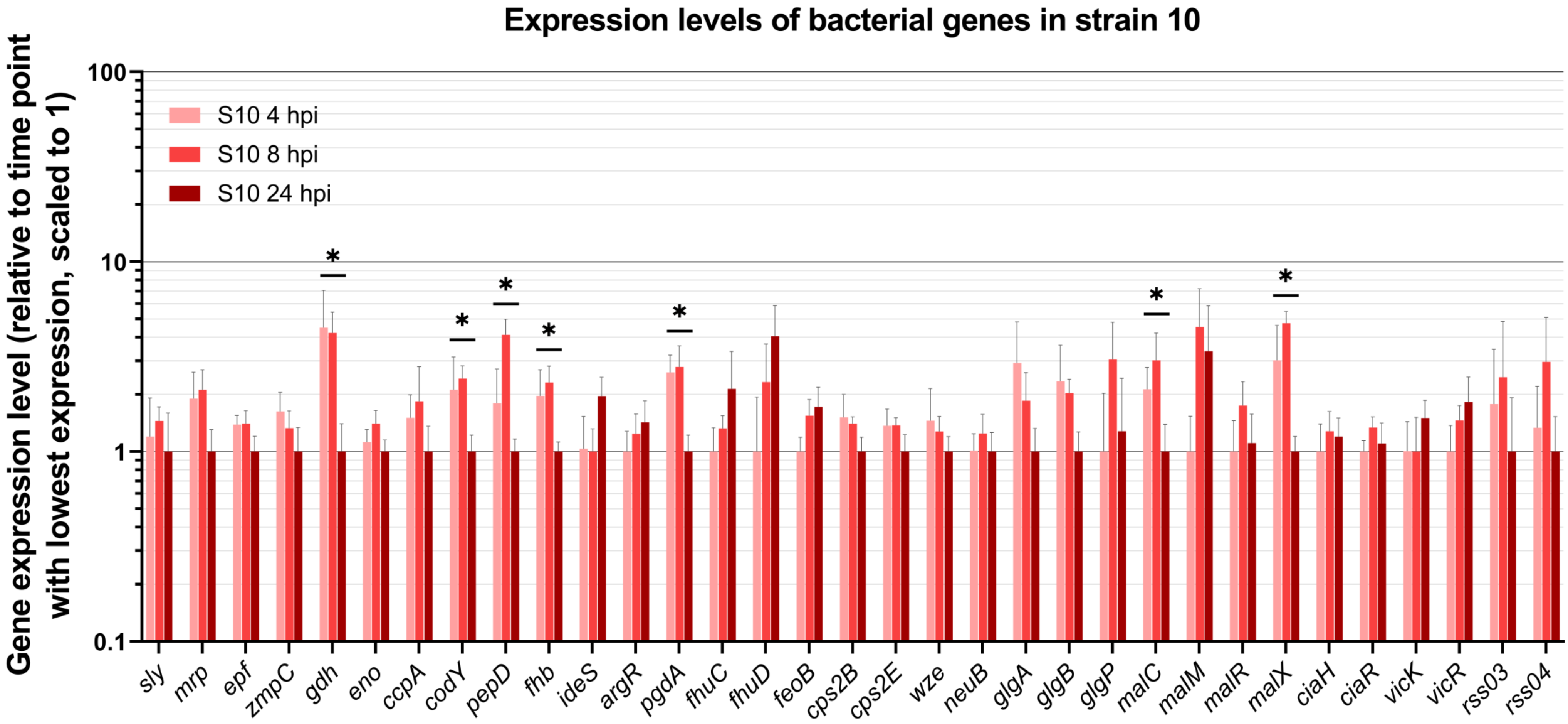
Expression levels of bacterial genes in strain 10
Gene expression level (relative to time point with lowest expression, scaled to 1)
100
10
1
0.1
S10 4 hpi
S10 8 hpi
S10 24 hpi
*
sly
mrp
epf
zmpC
gdh
eno
ccpA
codY
pepD
fhb
ideS
argR
pgdA
fhuC
fhuD
feoB
cps2B
cps2E
wze
neuB
glgA
glgB
glgP
malC
malM
malR
malX
ciaH
ciaR
vicK
vicR
rss03
rss04

Figure S6. PCA of bacterial gene expression in porcine PCLS from 4 to 24 hpi. PCA is based on the expression levels of all genes that were successfully quantified in all three *S. suis* strains. A) 4 hpi. B) 8 hpi. C) 24 hpi. Green – strain 8067. Blue – strain T15. Red – strain S10.

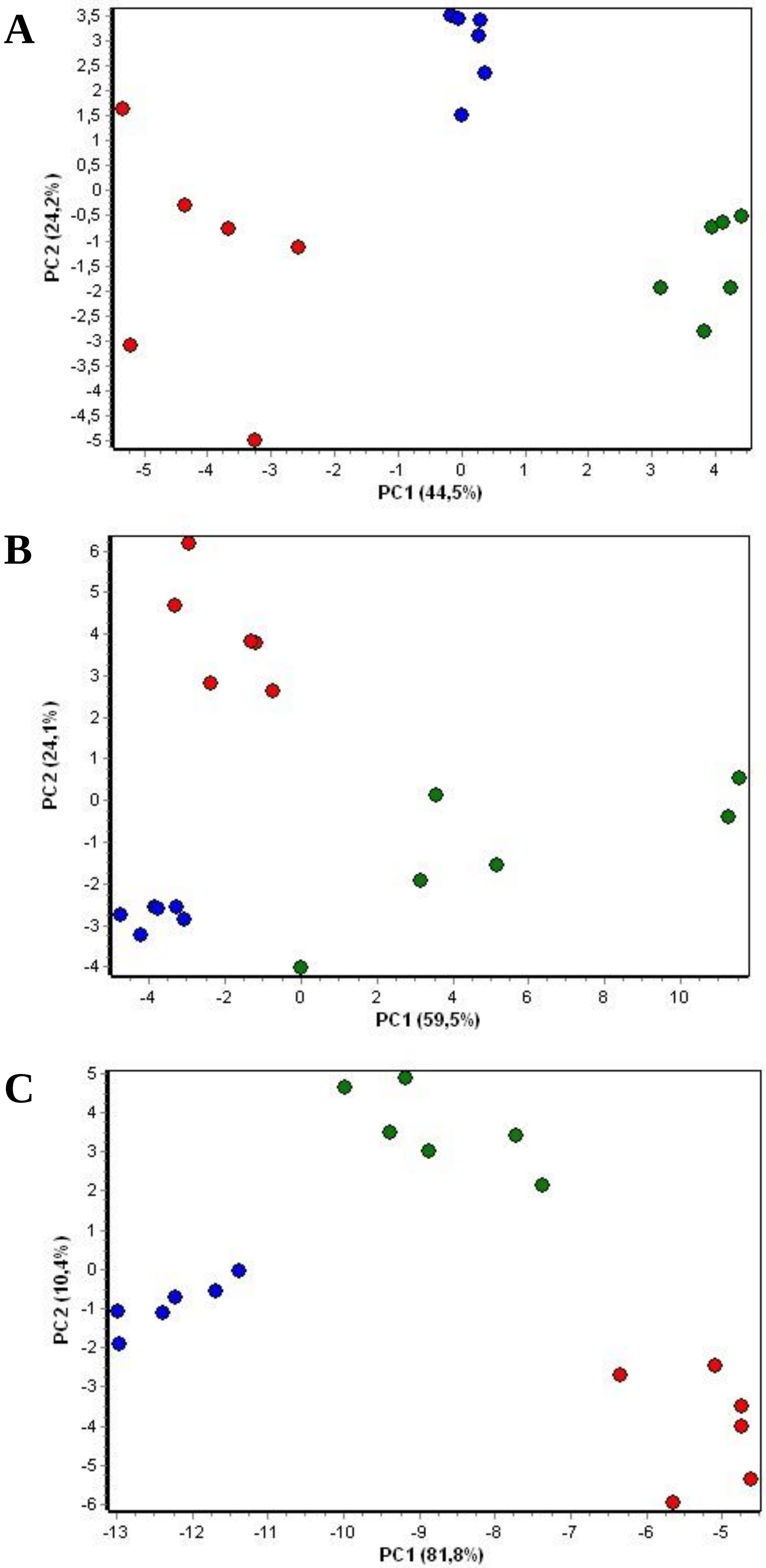

Figure S7. PCA of *S. suis* strains 8067, T15, and S10 gene expression in porcine PCLS. PCA is based on the expression levels of all genes that were successfully quantified in all three *S. suis* strains. A) strain 8067. B) strain T15. C) strain S10. White – 4 hpi. Grey – 8 hpi. Black – 24 hpi.

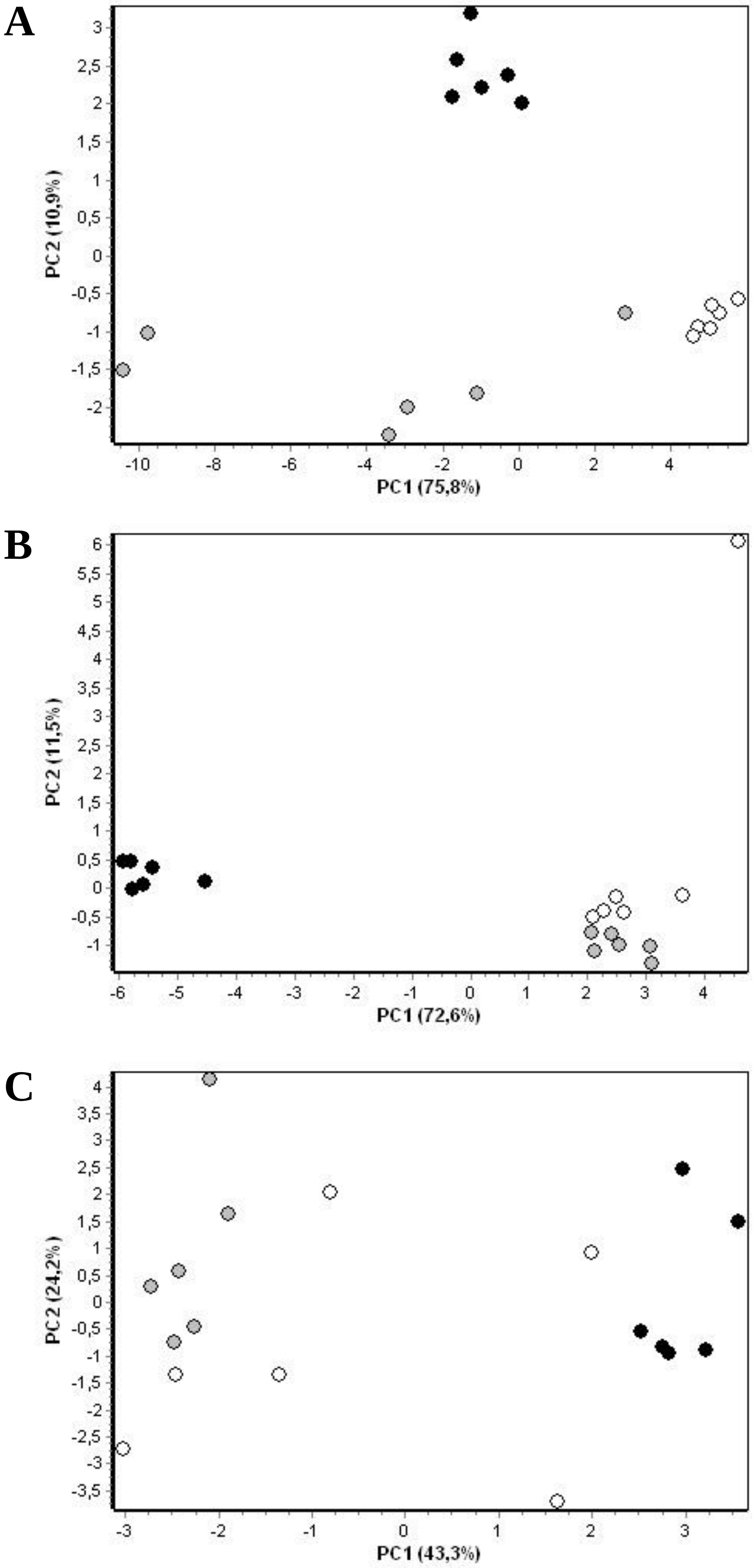

Supplement: Supplementary file 1 [file pathogens-13-00004-s001.zip › pathogens-2735810-supplementary.pdf]
